# Effect of Bionic Surface Architecture and Ultrasonic Power Density on Wear Resistance of Ni–AlN Nanoparticle-Reinforced Composite Coatings

**DOI:** 10.3390/ma19214552

**Published:** 2026-10-28

**Authors:** Hongbin Zhang, Shutong Yao, Chunlei Zhao, Rui Liu, Shuting Li, Chenglin Miao

**Affiliations:** 1 College of Mechatronic Engineering, Qiqihar University, Qiqihar 161006, China; 17673185309@163.com (S.Y.); 18946188793@163.com (R.L.); 18846603675@163.com (S.L.); 2 Technical Development Center, Qiqihar Vehicle Co., Ltd., Qiqihar 161002, China; zhaochunlei123@163.com; 3 Qiqihar Liming Gas Co., Ltd., Qiqihar 161041, China; 15898044248@163.com

**Keywords:** bionic surface, ultrasonic-assisted jet electrodeposition, Ni–AlN composite coating, ultrasonic power density, wear resistance

## Abstract

First, three bionic surface architectures (pit, hexagonal, and coupled) were compared to identify the optimal geometry; subsequently, the effect of ultrasonic power density (UPD, 0–35 W/cm^2^) on coating microstructure and dry-sliding wear performance was evaluated on the selected coupled structure. In this study, using dung beetle pit structures and many-banded krait hexagonal dorsal scales as bio-inspiration, pit-type (S1), hexagonal-type (S2), and coupled-type (S3) Ni–AlN nanocomposite coatings were deposited on AISI 1045 carbon structural steel by ultrasonic-assisted jet electrodeposition. The effect of ultrasonic power density (UPD, 0–35 W/cm^2^) on coating microstructure and dry-sliding wear performance was investigated by combining ANSYS finite element simulation with tribological experiments (ASTM G133). The coupled structure (S3) reduced peak contact stress by 45.8% and cumulative wear volume by 11.4%. At UPD = 25 W/cm^2^, AlN nanoparticles (43.6 nm) were uniformly dispersed, Ni grains were refined to 32.5 nm, and the coating achieved a microhardness of 794 ± 22 HV, a COF of 0.32 ± 0.01, and the lowest wear loss rate among the tested UPD conditions (approximately 0.025% mass loss). Hall–Petch analysis (R^2^ = 0.96) supports an important contribution of grain refinement to coating strengthening, with a smaller contribution from Orowan strengthening. This work provides a bio-inspired morphology–nanoparticle reinforcement approach for surface wear protection of carbon structural steel.

## 1. Introduction

Carbon structural steel is regarded as a foundational engineering material and has long occupied the largest share among basic structural materials in mechanical manufacturing, construction, the automotive industry, and heavy machinery. Owing to its balanced mechanical properties, convenient processability, and low cost, it is used in components ranging from high-speed gears in automotive transmissions and critical shafts in construction machinery to connectors in steel structures and load-bearing frames in commercial vehicles [1,2]. However, long-term engineering practice has revealed an inherent limitation: its wear resistance is relatively poor. In humid marine environments, dusty mining operations, or power-transmission components subjected to repeated alternating loads, the surface is prone to oxidative corrosion and spalling wear. This leads to progressive deterioration of dimensional accuracy, shortened service life, and in severe cases, equipment shutdown or even safety accidents [3,4,5]. Therefore, targeted surface modification of carbon structural steel to enhance wear and corrosion resistance and overall service reliability, and to facilitate its cost-effective substitution for expensive alloy steels, is of significant practical and industrial value [6,7,8,9]. The present study focuses primarily on dry-sliding tribological performance as a baseline condition; corrosion resistance and multi-phase wear environments are outside the scope of this work and will be addressed in future studies.

Nanocomposite coating technology offers an effective route to mitigate the surface deficiencies of carbon structural steel. By embedding nanoscale second-phase particles such as SiC, AlN, or TiN into a metallic matrix, a composite microstructure combining matrix load-bearing and reinforcement-phase strengthening is constructed [10,11,12]. These nanoparticles impart distinctive physical and chemical properties to the coating through their intrinsic characteristics. When applied to carbon structural steel, this approach can compensate for the surface-performance deficiencies of the substrate. In the present work, AlN was selected rather than SiC or TiN as the reinforcement phase for three reasons: (1) AlN possesses a high thermal conductivity, enabling efficient removal of interfacial frictional heat and reducing temperature rise during sliding; (2) AlN exhibits good chemical stability in Watts-type plating baths and does not undergo side reactions with bath constituents; and (3) the hardness of AlN (approximately 12 GPa) is moderate, unlike that of SiC (approximately 28 GPa), which tends to cause mechanical damage to the cathode surface during co-deposition. Hard nanoparticles represented by AlN improve coating hardness and resistance to plastic deformation through Orowan strengthening and grain refinement, thereby enhancing wear resistance.

The selection of an appropriate deposition method is critical for producing high-quality nanocomposite coatings. Compared with conventional direct-current electroplating and ordinary jet electrodeposition, ultrasonic-assisted jet electrodeposition offers clear advantages in nanoparticle dispersion, deposition rate, and coating compactness, and is particularly suited to Ni-based composite coatings containing nanoscale second phases [13,14,15]. This technique is an effective and promising approach that combines two dynamic physical fields—ultrasonic vibration and jet flow. First, the cavitation effect induced by ultrasound is distinctive: cavitation bubbles undergo expansion, oscillation, and instantaneous collapse in a periodic acoustic pressure field, generating transient high-pressure microjets and intense agitation that break up nanoparticle agglomerates in the electrolyte and ensure uniform dispersion. The cavitation impact also effectively removes adsorbed hydrogen bubbles and impurity particles from the substrate surface, reducing pinholes and porosity, and can activate the substrate surface, thereby strengthening the interfacial bonding between the coating and the steel substrate. Second, the high-speed jet impinges directly on the cathode surface, sharply reducing the diffusion-layer thickness and accelerating mass transfer of ions and particles. This increases both deposition rate and current efficiency and yields a coating matrix with finer grains and denser structure.

Integrating biomimetic principles into surface engineering provides a design strategy for addressing high frictional resistance and severe wear in carbon structural steel under harsh tribological conditions. Natural evolution over hundreds of millions of years has produced numerous microstructures that are effective at reducing drag and wear [16,17,18]. When moving through soil, the dung beetle relies on a composite structure of micrometer-scale pits and fine grooves on its dorsal surface: these break the continuity of fluid flow to form a turbulent buffer layer for drag reduction, while also distributing abrasive-particle pressure, reducing the actual contact area, and combining with a surface wax layer to significantly reduce frictional loss [19,20]. Snakes crawling on rough ground possess overlapping, imbricate scales and directional microtextures that guide fluid flow and suppress turbulence to reduce adhesive resistance on the one hand, and provide a hard protective layer, stress distribution, and impact buffering on the other [21]. These observations suggest that surface modification need not rely solely on the intrinsic properties of the material; active design and construction of specific micro–nano geometric morphologies or multi-scale composite structures can yield surface performance exceeding that of conventional homogeneous materials. This “structure-enabled” approach may be key to overcoming performance bottlenecks.

Jet electrodeposition is an efficient method for preparing nanoparticle-reinforced composite coatings and has accumulated a substantial body of research on deposition mechanisms and performance characterization in the field of metallic surface modification [22,23,24]. Existing studies fall into two categories: (i) laser surface texturing combined with PVD or electroless plating; and (ii) jet electrodeposition on smooth flat surfaces. Existing studies on ultrasonic-assisted jet electrodeposition have mainly focused on process optimization and basic performance of coatings on smooth substrates, while the coupling of this technique with biomimetic surface topologies—particularly the synergistic effects of geometric stress redistribution, debris trapping, and nanoparticle strengthening in Ni–AlN systems—remains largely unexplored.

In this work, jet electrodeposition and biomimetic principles are combined. Bionic micro–nano structures are first fabricated on the surface of carbon structural steel by precision machining, followed by deposition of a Ni–AlN nanocomposite coating, forming a dual synergistic reinforcement system of “bionic micro–nano morphology plus nanoparticle reinforcement.” The influence of different ultrasonic power densities on the microstructure, interfacial bonding, and tribological properties of the composite coating is investigated. The novel contributions of this study are as follows: (1) the UPD–microstructure–wear-resistance relationship is quantified for the first time in a bionic-textured-substrate/Ni–AlN system; (2) the secondary agglomeration mechanism of AlN particles at 35 W/cm^2^ (SDS desorption → loss of electrostatic repulsion → collision-induced agglomeration) is revealed and is supported by EDS analysis of the wear surface; and (3) a process window at UPD = 25 W/cm^2^ is identified and quantitatively correlated with the Hall–Petch relationship.

Based on the above analysis, the following hypotheses were proposed: (i) the coupled pit–hexagonal texture reduces dry-sliding wear relative to single-structure textures through a synergistic mechanism of macroscopic stress distribution and microscopic wear-debris management; (ii) there exists an appropriate UPD window within the tested range that simultaneously maximizes AlN dispersion and Ni grain refinement; and (iii) beyond this window, excessive ultrasonic power induces secondary agglomeration of AlN particles, leading to degradation of coating performance.

## 2. Materials and Methods

### 2.1. Material Selection and Specimen Preparation

Cathode specimens were made of AISI 1045 carbon structural steel (equivalent to Chinese GB/T 699 45# steel), with a nominal chemical composition (wt%) of 0.42–0.50 C, 0.17–0.37 Si, 0.50–0.80 Mn, ≤0.045 S, and ≤0.045 P. The steel was supplied in the normalized condition, with a measured baseline surface hardness of 192 ± 5 HV and a Young’s modulus of approximately 210 GPa. This value is substantially lower than the hardness of the deposited coatings (536–794 HV). The deformation compatibility of a thin coating on a soft substrate under a 5 N load was considered: under the present experimental conditions, the substrate underwent predominantly elastic deformation, and no pronounced plastic deformation affecting coating lifetime was observed. AISI 1045 steel is widely used in mechanical manufacturing and provides a stable substrate for electrodeposition. The anode was a nickel rod of 99.8% purity and 500 mm length; high-purity nickel ensures uniform anodic dissolution and minimizes impurity incorporation. The inter-electrode gap was set to 5 mm, which ensures uniform electric-field distribution while avoiding obstruction of electrolyte flow.

The AlN nanoparticle concentration was fixed at 8 g/L, a value optimized based on preliminary experiments and the literature [25]. Preliminary concentration-gradient tests (2–14 g/L) showed that below 5 g/L the reinforcement effect was insignificant due to insufficient particle content, while above 12 g/L severe particle agglomeration degraded coating quality. The hardness peaked at 713 ± 24 HV and the wear loss rate reached a minimum of 0.026 ± 0.002% at 8 g/L, which was selected as the equilibrium point that maximized particle codeposition while maintaining good dispersion. All reagents were of analytical grade and purchased from Sinopharm Chemical Reagent Co., Ltd. (Shanghai, China). The AlN nanoparticles used in this study had a purity of ≥99.9%, a mean particle size of approximately 43.6 nm (confirmed by TEM, Section 3.2), and a hexagonal wurtzite crystal structure. Prior to deposition, the AlN powder was dispersed in the plating solution by ultrasonication for 30 min to break up soft agglomerates. Table 1 lists the chemical composition and operating parameters of the plating bath, which were determined based on relevant literature and optimization under the present experimental conditions.

**Figure 1:**
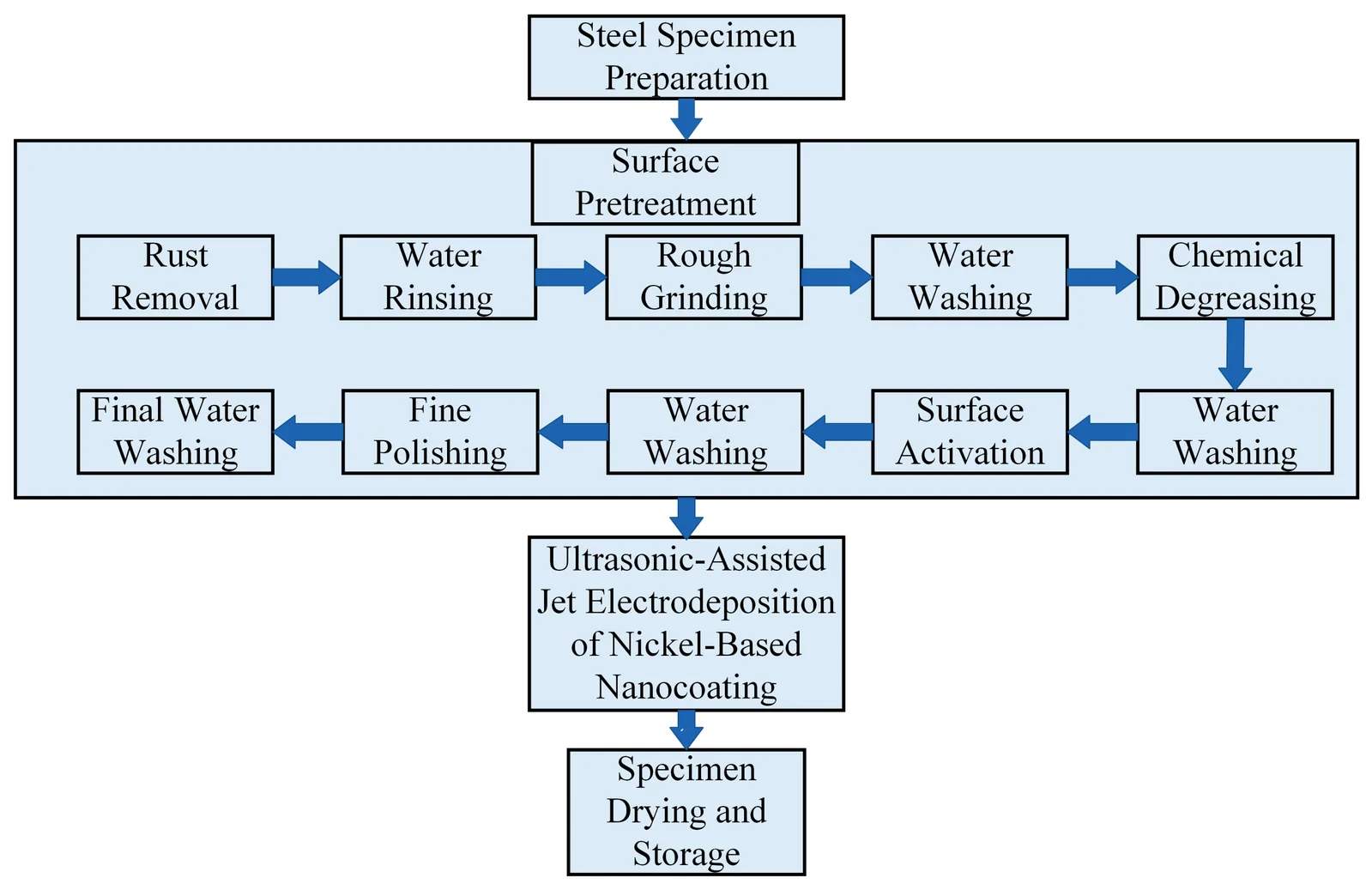
Pretreatment process flow diagram for plating preparation.

Prior to ultrasonic-assisted pulse jet electrodeposition, specimen pretreatment was essential. The surfaces were sequentially ground with 100#, 150#, 240#, 320#, and 1000# SiC abrasive papers in a strictly coarse-to-fine sequence to avoid deep scratches from skip-grinding. The specimens were then chemically degreased in a 15 g/L NaOH solution at 45 °C for 20 min; this temperature rapidly dissolves surface oil without excessive etching of the substrate. This was followed by activation in a 1 mol/L HCl solution for 1 min to remove surface rust and oxide films. Finally, the specimens were thoroughly rinsed with deionized water. The pretreatment flow is illustrated in Figure 1.

**Figure 2:**
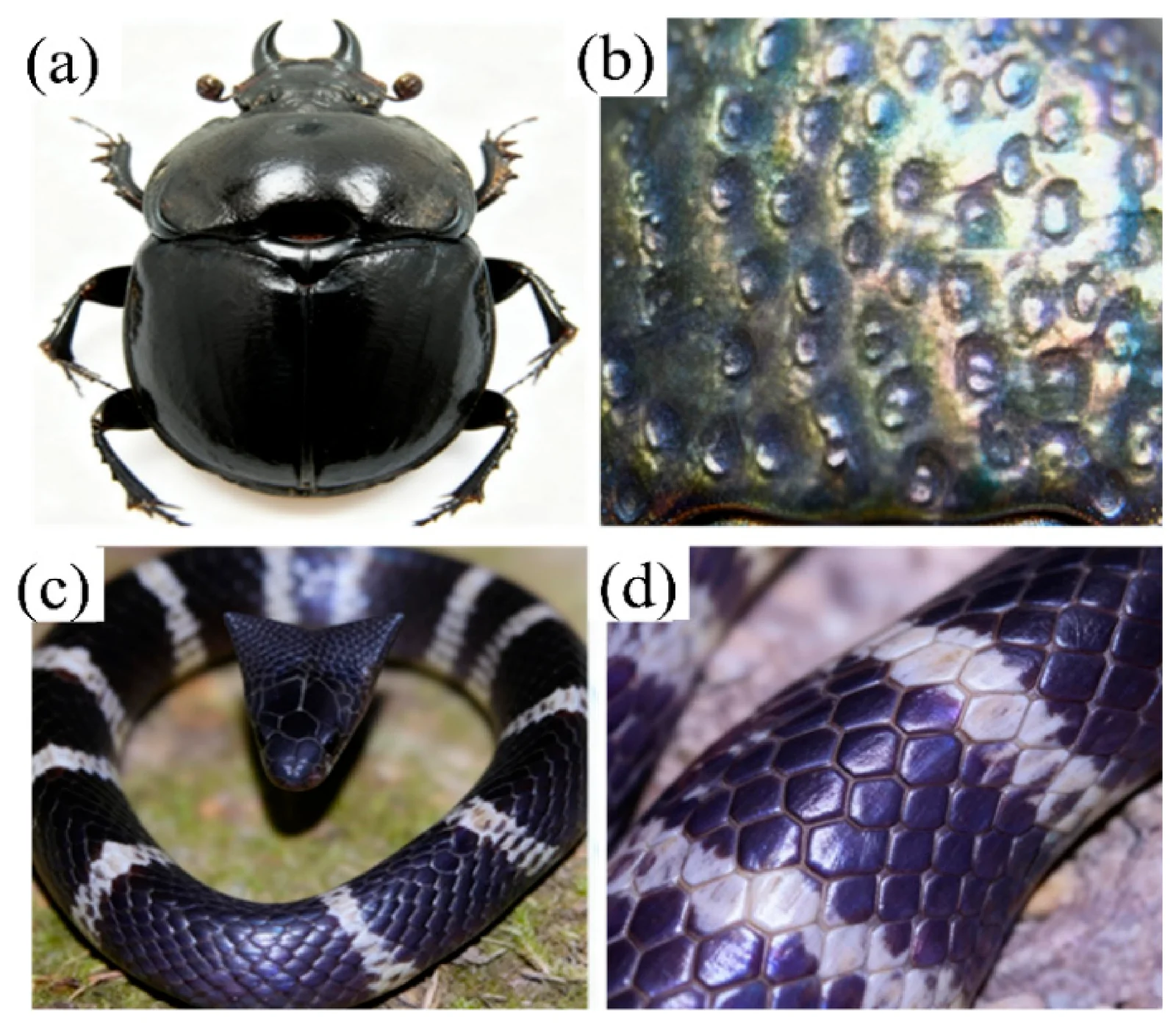
Biological prototypes: (**a**) dung beetle body (scale bar: 5 mm); (**b**) enlarged view of head pits (scale bar: 1 mm); (**c**) many-banded krait body (scale bar: 10 mm); (**d**) enlarged view of dorsal hexagonal scales (scale bar: 500 μm).

In nature, the body surfaces of many organisms have evolved micro–nano structures with pronounced friction-reducing and wear-resistant characteristics. The pit structures on the dung beetle were selected because they evolved in a soil environment containing hard abrasive particles and serve three functions: (i) accommodating soil particles to reduce three-body abrasion, (ii) reducing the actual contact area, and (iii) creating a non-smooth surface that minimizes adhesion. These functions directly correspond to the objectives of dry-sliding wear and debris management in the present study. The hexagonal scale pattern of snakes was selected because, during crawling on rough substrates, the scales function to (i) distribute contact stress and prevent local indentation, (ii) guide stress diffusion along scale edges, and (iii) provide a wear-resistant surface layer of hard keratin. These structures are illustrated in Figure 2. The geometric parameters were scaled from the natural dimensions of dung beetle elytron pits (100–300 μm) and krait dorsal scale hexagons (50–150 μm) reported in biological studies, and were optimized for the present coating thickness range (35–60 μm) via preliminary tribological tests. The pit diameter was 100 μm and the spacing was 200 μm, designed proportionally within the measured range of dung-beetle head pits (100–300 μm) reported in the literature. The hexagonal cell side length of approximately 80 μm was determined by preliminary orthogonal experiments using coefficient of friction and mass loss as evaluation indices.

**Figure 3:**
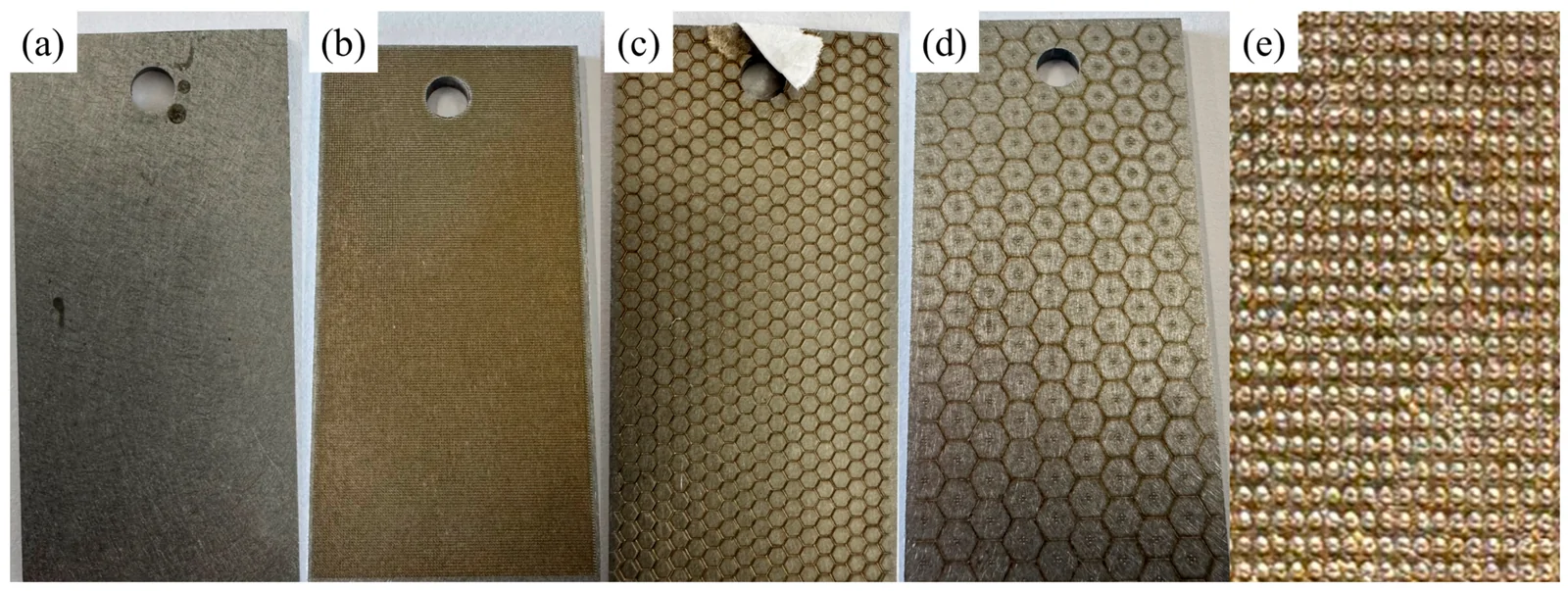
Optical photographs of specimen surfaces: (**a**) smooth; (**b**) hexagonal texture; (**c**) pit texture; (**d**) coupled pit–hexagonal texture; (**e**) enlarged view of pit texture.

Three bionic structures were designed: a pit structure (pit diameter approximately 100 μm, depth approximately 20 μm, center-to-center spacing approximately 200 μm, arranged in a hexagonal close-packed pattern), a hexagonal structure (hexagonal cell side length approximately 80 μm, wall thickness approximately 20 μm), and a coupled structure combining both pit and hexagonal features. All textured surfaces were fabricated by precision micro-milling using a CNC micromachining system (M400, Kern Microtechnik GmbH, Murnau, Germany) with a 100 μm diameter solid carbide end mill for pit fabrication and a 20 μm diameter solid carbide micro-end mill for the hexagonal grid, spindle speed 30,000 rpm, feed rate 5 mm/s, and depth of cut 5 μm per pass. The geometric parameters were verified by optical profilometry (Contour GT-X, Bruker Corporation, Billerica, MA, USA) with a measurement uncertainty of ±0.5 μm. The pit structure had a texture area fraction of approximately 22%. Specimens were oriented with the hexagonal grid edges aligned parallel to the sliding direction. Three-dimensional solid models were constructed in SolidWorks 2022 (Dassault Systèmes, Vélizy-Villacoublay, France). Figure 3 shows photographs of specimens with smooth, hexagonal, pit, and coupled textures.

The total electrolyte volume in the plating tank was 2 L. The jet nozzle had an inner diameter of 1.5 mm, the nozzle-to-cathode distance was maintained at 5 ± 0.5 mm, and the electrolyte flow rate was controlled at 2.0 ± 0.1 L/min, corresponding to a jet velocity of approximately 19 m/s at the nozzle exit. The nozzle was aligned normal to the specimen surface and scanned horizontally at 2 mm/s with a stroke width of 15 mm, covering the entire exposed cathode area of 40 mm × 13 mm. The nominal current density was 3 A/dm^2^, pulse frequency 100 kHz, duty cycle 40%, bath temperature 45 ± 1 °C, and pH 5.5.

Jet electrodeposition is a technique in which electrolyte is directed onto the cathode surface by a high-speed jet. The molar deposition rate Rd (mol·s^−1^) can be expressed as Equation (1):

(1) $$R_{d}=\frac{j\cdot A\cdot \eta }{nF}$$

where j is current density, A is deposition area, η is current efficiency, z is the electron transfer number, and F is Faraday’s constant (the per-unit-area deposition rate is jη/(zF)). The corresponding linear deposition rate can be derived by multiplying by the molar mass of Ni (58.69 g·mol^−1^) and dividing by the coating density (~8.9 g·cm^−3^). Strengthening in the Ni–AlN composite coating arises primarily from grain refinement (Hall–Petch effect): the grain size d and hardness H satisfy:

(2) $$H=H_{0}+kd^{-\frac{1}{2}}$$

where H0 and k are material constants.

**Figure 4:**
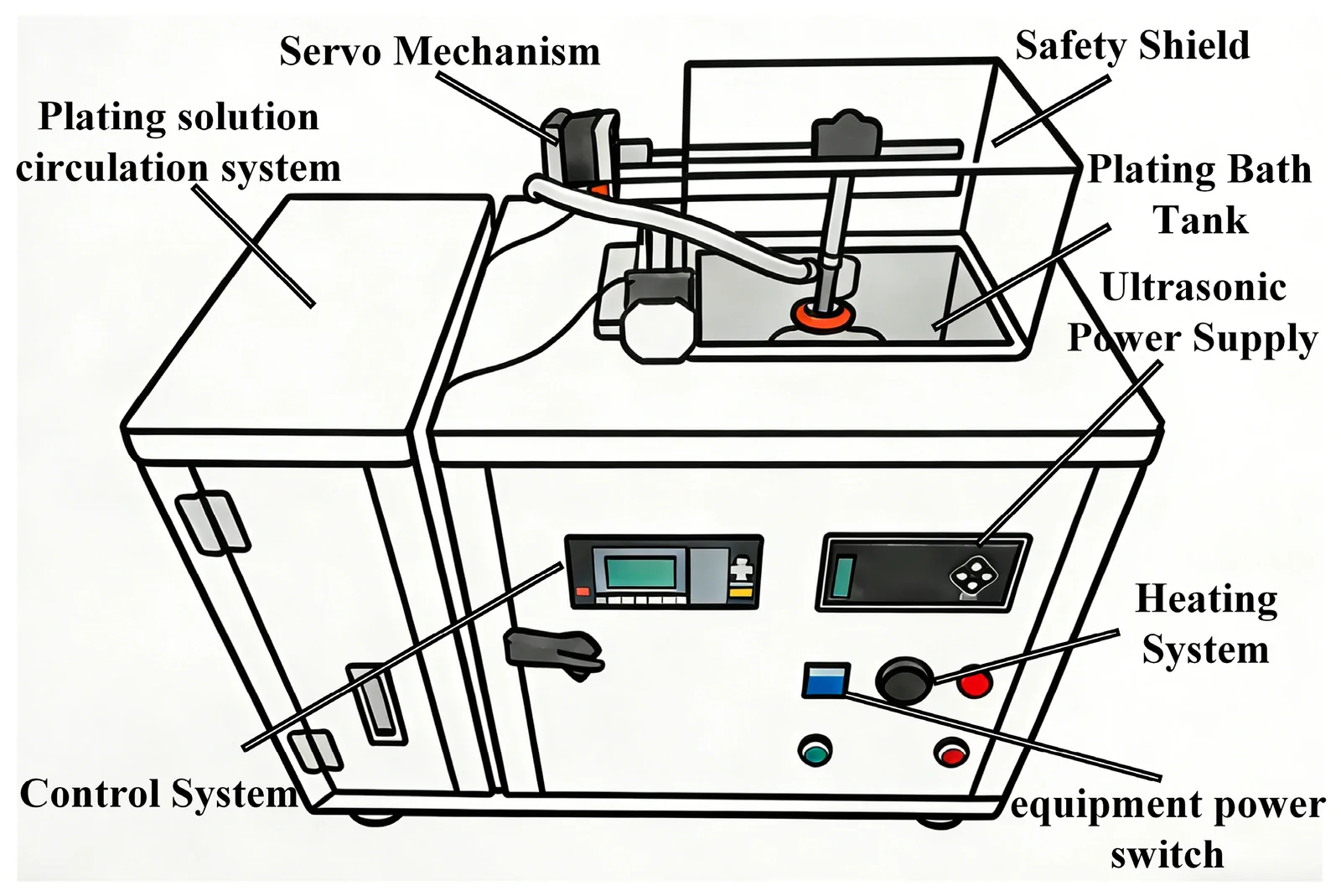
Schematic diagram of the ultrasonic-assisted jet electrodeposition experimental setup.

The experimental device consists of a plating solution circulation system, protective cover, plating solution tank, ultrasonic generator, control system, heating system, servo mechanism and other components. The experimental equipment is shown in Figure 4. The ultrasonic system consisted of an ultrasonic generator (KQ-200VDE, Kunshan Ultrasonic Instrument Co., Kunshan, Jiangsu, China) operating at a fixed frequency of 20 kHz, connected to a titanium alloy transducer immersed in the plating bath. The effective radiating area of the transducer was approximately 50 cm^2^. The transducer was placed 50 mm directly below the cathode specimen, with the plating solution depth maintained at 80 mm. The reported ultrasonic power density (UPD) values of 0, 15, 25, and 35 W/cm^2^ were calculated as the nominal electrical output power divided by the effective radiating area of the transducer. The actual acoustic intensity was calibrated using the calorimetric method: the temperature rise in the plating solution over 5 min was measured, and the dissipated acoustic power was computed from the heat balance equation. The measured acoustic intensity agreed with the nominal values within ±10%. All reported UPD values correspond to the nominal electrical input power density normalized by the transducer radiating area.

**Figure 5:**
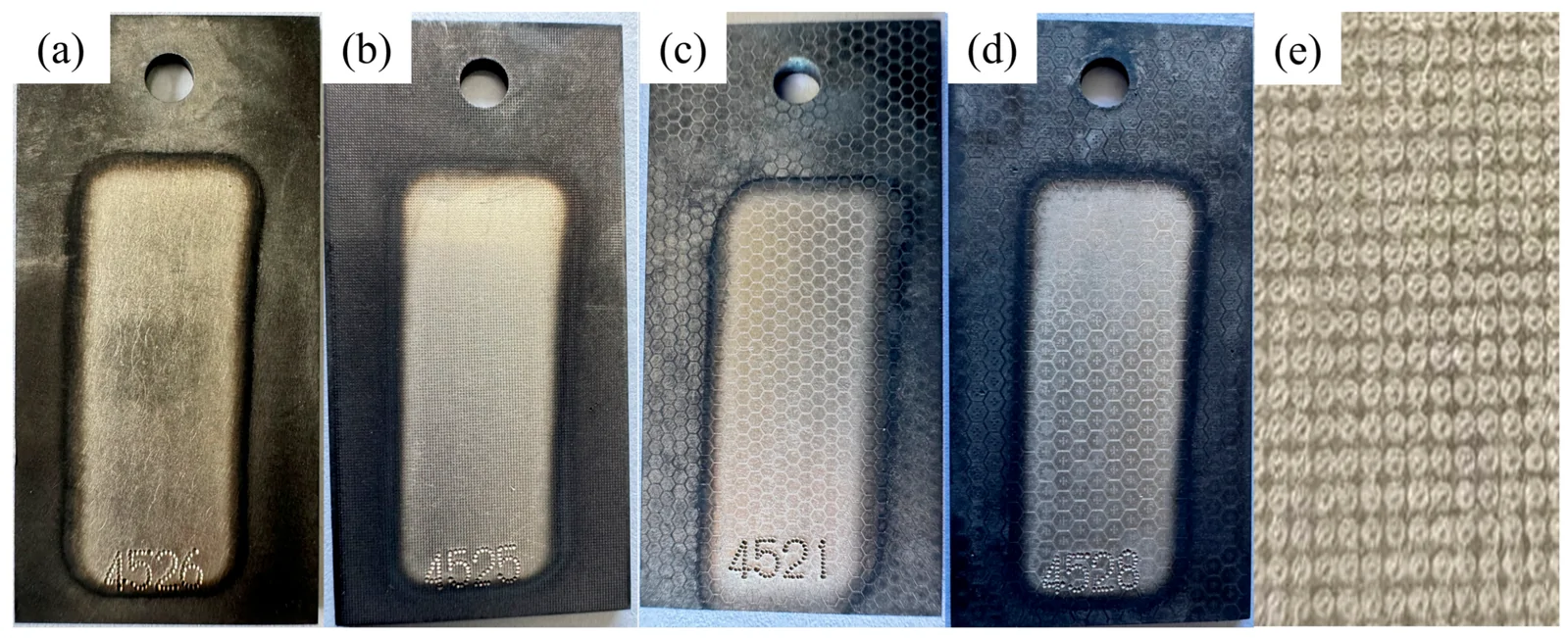
Physical photographs of Ni–AlN composite coatings on specimens with different bionic structures: (**a**) smooth; (**b**) pit; (**c**) hexagonal; (**d**) coupled; (**e**) Biomimetic pit magnified image.

The physical specimens of Ni–AlN nanoparticle-reinforced composite coatings prepared by this equipment are shown in Figure 5.

### 2.2. Characterization and Tribological Testing

To comprehensively and quantitatively evaluate the Ni–AlN nanoparticle-reinforced composite coatings prepared under different UPD conditions, a series of characterization techniques was employed. All friction and wear tests were conducted at room temperature under dry-sliding conditions (no lubrication, no corrosive medium, no external abrasive particles). At least three parallel specimens were tested for each parameter to avoid random errors. All quantitative results are reported as mean ± standard deviation (SD), with *n* = 3. Error bars in the figures represent ± SD. Tribological tests were conducted following the guidelines of ASTM G133 (standard test method for linearly reciprocating ball-on-flat sliding wear). All tribological tests were conducted for a total duration of 30 min per specimen using an MDW-02 high-speed reciprocating friction and wear tester. The tribopair consisted of a GCr15 steel ball of 6 mm diameter and a flat specimen. Test parameters were: normal load 5 N, sliding speed 0.1 m/s, reciprocating stroke 5 mm. Mass loss was determined using an electronic analytical balance (ME204E, Mettler-Toledo International Inc., Greifensee, Switzerland) with a precision of 0.1 mg. All tests were conducted at room temperature (25 ± 2 °C) and relative humidity of 45 ± 5%. The wear loss rate was calculated as the percentage of mass loss relative to the initial coating mass:

(3) $$\left(\mathrm{Wear}\;\mathrm{loss}\;\mathrm{rate}\;(\%)=\frac{m_{\mathrm{before}}-m_{\mathrm{after}}}{m_{\mathrm{coating}}}\times 100\%\right)$$

where m_before_ and m_after_ are the specimen masses before and after the wear test, and m_coating_ is the calculated coating mass based on average thickness and density. Each value is reported as mean ± SD (*n* = 3). We note that the analytical balance used has a resolution of 0.1 mg; the coating mass losses (on the order of a few mg for the thinner coatings) are close to this resolution limit, and the reported wear-loss rates should be interpreted with this uncertainty in mind.

Microhardness tests followed ASTM E384 with a load of HV0.1 and a dwell time of 15 s. Surface morphology was observed by scanning electron microscopy (SEM, ZEISS Sigma 300, Carl Zeiss AG, Oberkochen, Germany). Prior to observation, specimens were gold-sputtered for approximately 30 s to enhance conductivity without obscuring the true surface topography. Cross-sectional specimens were prepared by mechanical grinding with SiC papers up to 2000 grit, followed by polishing with 1 μm diamond paste. EDS point analysis was performed using an Oxford Instruments X-Max 80 detector at 15 kV. Quantification was performed using standardless ZAF correction, with the gold sputter coating contribution accounted for during data processing.

Quantitative surface roughness was evaluated by atomic force microscopy (AFM, Bruker Dimension Icon, Bruker Nano GmbH, Berlin, Germany) in tapping mode using a silicon probe (RTESPA-300, Bruker, tip radius < 12 nm). Scan size was 50 × 50 μm^2^, with 512 × 512 pixels. Three independent regions were measured on each specimen. Raw images were subjected to first-order flattening to remove sample tilt. The arithmetic mean roughness (Ra) and root-mean-square roughness (Rq) were calculated over the full scan area, and reported as mean ± SD (*n* = 3). To reveal the nanoscale microstructure, the coating prepared at UPD = 25 W/cm^2^ was characterized by transmission electron microscopy (TEM, JEM-2100F, JEOL Ltd., Akishima, Tokyo, Japan). Thin foil specimens were prepared by mechanical grinding followed by ion milling. High-resolution TEM (HRTEM) was used to observe lattice fringes of AlN nanoparticles, and selected area electron diffraction (SAED) was used to identify the crystalline phase.

Microhardness was measured at HV0.1 with a 15 s dwell time, taking the average of eight randomly selected points on each specimen. The indentation depth was approximately 2–3 μm, well below the minimum coating thickness, ensuring that the measured values reflect coating properties rather than substrate influence. Regarding hydrogen embrittlement: pulse electrodeposition generates significantly less hydrogen than direct-current electroplating, and the high-speed jet continuously flushes the cathode surface, reducing hydrogen-bubble retention. No post-deposition baking was performed in this study. It should be noted that for thin coatings (approximately 50 μm) and short-duration wear tests (30 min), the influence of hydrogen embrittlement may be limited on the present experimental time scale; however, hydrogen embrittlement under long-term service conditions will be investigated in future work using slow-strain-rate tensile testing.

### 2.3. Finite Element Simulation

ANSYS is a widely used engineering simulation platform capable of coupling structural, fluid, and electric fields for multi-physics analysis of solid models, providing strong support for numerical simulation of friction and wear.

The Archard wear equation [26] was used as the basis for establishing a wear model under dry-sliding conditions. Equation (4) shows the quantitative relationship among wear volume, material hardness, sliding distance, and applied load:

(4) $$V=K\frac{\mathrm{Fs}}{H}$$

where V is wear volume, F is normal load, s is sliding distance, H is the hardness of the softer material, and K is the wear coefficient. All finite element simulations were performed using ANSYS Workbench 2021 R2 (ANSYS Inc., Canonsburg, PA, USA) with the transient structural module. Four models—smooth (S0), pit (S1), hexagonal (S2), and coupled (S3)—were meshed with 10-node quadratic tetrahedral elements (SOLID187). The contact region between the upper and lower sliders was refined to an element size of 25 μm, while the bulk regions used a coarser mesh of approximately 200 μm. Total element counts were approximately 180,000 (S0), 220,000 (S1), 250,000 (S2), and 280,000 (S3). Mesh convergence was verified by refining the contact mesh from 50 μm to 25 μm, which changed peak contact stress and cumulative wear volume by less than 3%. Surface-to-surface contact with an Augmented Lagrange formulation was defined between the two bodies, with a friction coefficient corresponding to each experimental condition. The Archard wear model was implemented with K = 1.5 × 10^−3^ mm^3^/(N·m) and a pressure exponent C_3_ = 1. The maximum wear increment per substep was limited to 0.5 μm, and wear geometry was updated automatically at each time step. The time step was set to 0.01 s with automatic time stepping enabled.

(5) $$\mathrm{Ah}=K\frac{\mathrm{Fs}}{H}$$

where A is the actual contact area and h is the wear depth. Solving yields:

(6) $$h=K\frac{\sigma s}{H}$$

where σ is the normal contact stress (Pa) during sliding. Wear accumulates with sliding distance, which is the product of sliding velocity and time (v = ds/dt). Differentiation gives Equation (7):

(7) $$\frac{\mathrm{dh}}{\mathrm{dt}}=K\frac{\sigma v}{H}$$

Integrating Equation (7) yields the theoretical wear-depth model (Equation (8)):

(8) $$h=K\int \frac{\sigma v}{H}\mathrm{dt}$$

(τ=μ·σ) To simplify the calculation, the ball-on-flat contact was approximated as a flat-on-flat contact. According to Hertzian contact theory, a GCr15 steel ball of 6 mm diameter in contact with a flat specimen under a 5 N normal load produces a maximum compressive contact stress of approximately 1.13 GPa and a contact radius of approximately 0.046 mm. This macroscopic normal pressure is substantially higher than the frictional shear stresses shown in Figure 7, which scale with the coefficient of friction (μ). The equivalent uniformly distributed load applied in the simulation was calibrated to match the average Hertzian contact pressure, producing the observed shear stress distribution. The reduced elastic modulus E* was calculated as E/[2(1 − ν^2^)] = 115.4 GPa, accounting for the elastic deformation of both the ball and the flat specimen. In the simulation, an equivalent uniformly distributed load was applied to the upper slider so that the average contact-surface stress matched the experimental Hertzian contact stress, ensuring comparability. It should be noted that this Hertzian estimate is based on ideal half-space assumptions and is used only to calibrate the equivalent distributed load in the finite element model; the exact stress distribution, including stress concentration at texture edges, is obtained from the FEA results rather than from analytical Hertz theory.

An 8 mm stroke was used in the simulation to obtain fully developed stress fields and sufficient wear accumulation for reliable comparison. The experimental stroke of 5 mm is shorter, but key comparative metrics (peak stress, steady-state friction, wear rate per unit distance) are stroke-independent for established steady-state sliding, so this difference does not affect the qualitative comparison or trend validation. The model was imported into ANSYS Workbench with the lower slider fixed and the upper slider moving linearly along the x-direction over an 8 mm stroke. The upper slider was assigned GCr15 steel properties (elastic modulus 210 GPa, Poisson’s ratio 0.3), and the lower slider was assigned AISI 1045 steel properties (elastic modulus 210 GPa, Poisson’s ratio 0.3). The coating region was assigned the same material properties as the substrate to isolate the purely geometric effect of the bionic textures. This is a deliberate simplification: the model evaluates how surface morphology redistributes contact stress and influences wear, but does not capture the additional strengthening effect of the Ni–AlN coating itself. Therefore, simulation results validate the geometric contribution to wear reduction, not the full coated-system performance.

**Figure 6:**
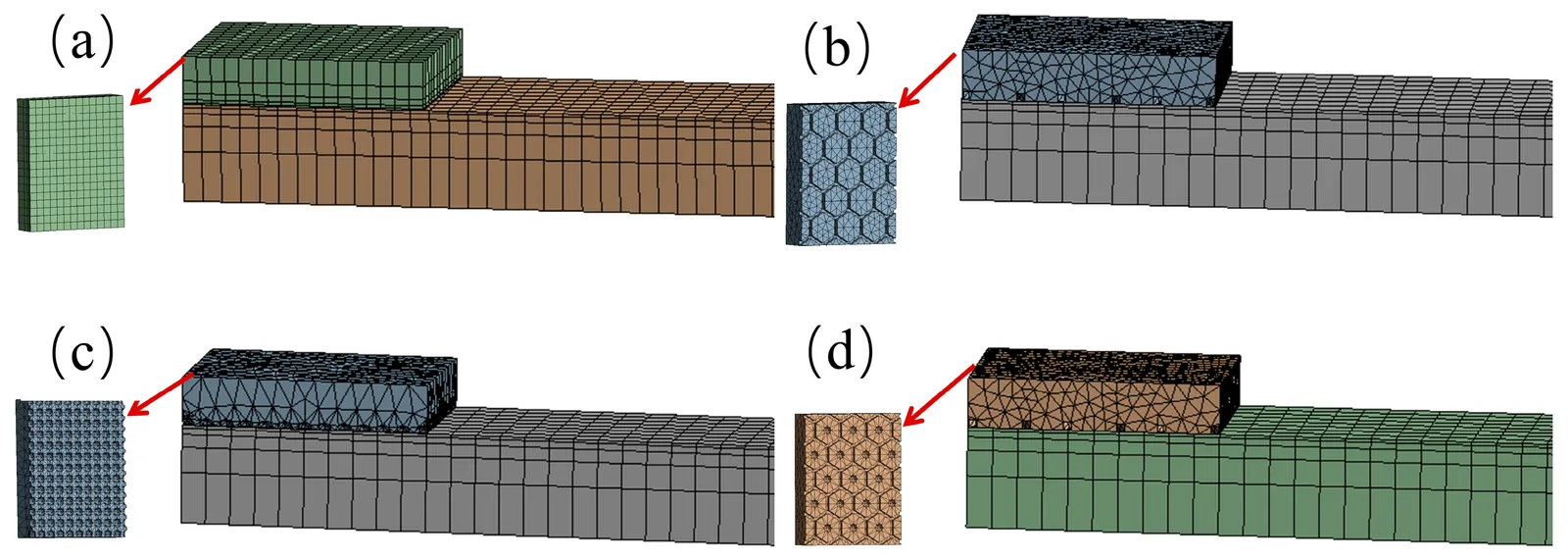
Finite element meshing diagrams: (**a**) S0 smooth; (**b**) S1 pit; (**c**) S2 hexagonal; (**d**) S3 coupled.

$\left(q=\mu \cdot p\cdot v\right)$ The sliding velocity was set to 0.1 m/s, consistent with the experimental average. The coefficient of friction was taken from experimental measurements (S0 = 0.54, S3 = 0.39). The meshed models are shown in Figure 6. Temperature simulations were performed under transient conditions with an initial uniform temperature of 25 °C. Frictional heat generation was modeled as a surface heat flux at the contact interface, calculated as q = μ·p·v. A heat partition coefficient of 0.5 was assumed between the two contacting bodies. Thermal properties used were: thermal conductivity 50 W/(m·K), specific heat 470 J/(kg·K), and density 7850 kg/m^3^ for steel. Convective heat loss to ambient air was applied to all free surfaces with a heat transfer coefficient of 10 W/(m^2^·K). Radiation heat loss was neglected given the small temperature rise.

## 3. Results

### 3.1. Structural Simulation Analysis

**Figure 7:**
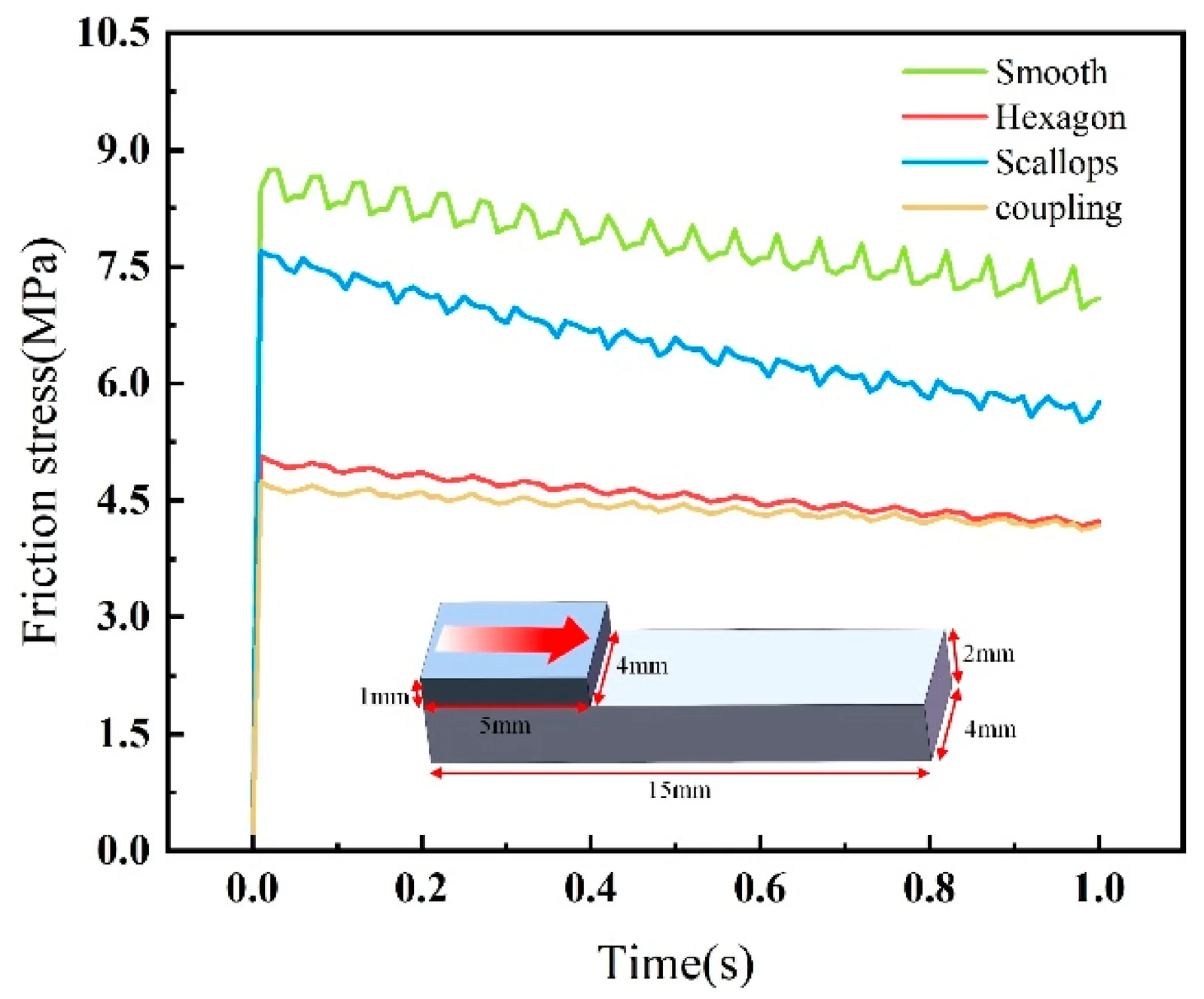
Frictional shear stress distribution over sliding time for different bionic surfaces.

The peak frictional shear stresses on the contact surfaces, obtained from ANSYS Workbench simulations, are shown in Figure 7. The maximum shear stresses for S0, S1, S2, and S3 were 8.749 MPa, 7.699 MPa, 5.065 MPa, and 4.744 MPa, respectively, showing a monotonically decreasing trend. S0 exhibited the highest shear stress because the smooth surface concentrated contact pressure over an extremely small area, with no geometric feature to distribute the stress. S1 reduced the stress through geometric stress redistribution: the pits transferred contact pressure from sharp asperities to a broader region, achieving local stress relief, though with lower efficiency than the regular S2 arrangement. S2 further reduced the stress through a periodic grid-like arrangement that distributed load uniformly across multiple support points and guided stress diffusion along edges and vertices, approaching an equal-strength design. S3 achieved the lowest shear stress through the synergistic action of both structures: the pits first distributed part of the pressure at the micro scale, and the hexagonal grid then further redistributed the dispersed pressure over a larger area, forming a coupled mechanism of “micro-scale dispersion plus macro-scale guidance” that produced the most uniform contact-stress distribution among the tested conditions.

**Figure 8:**
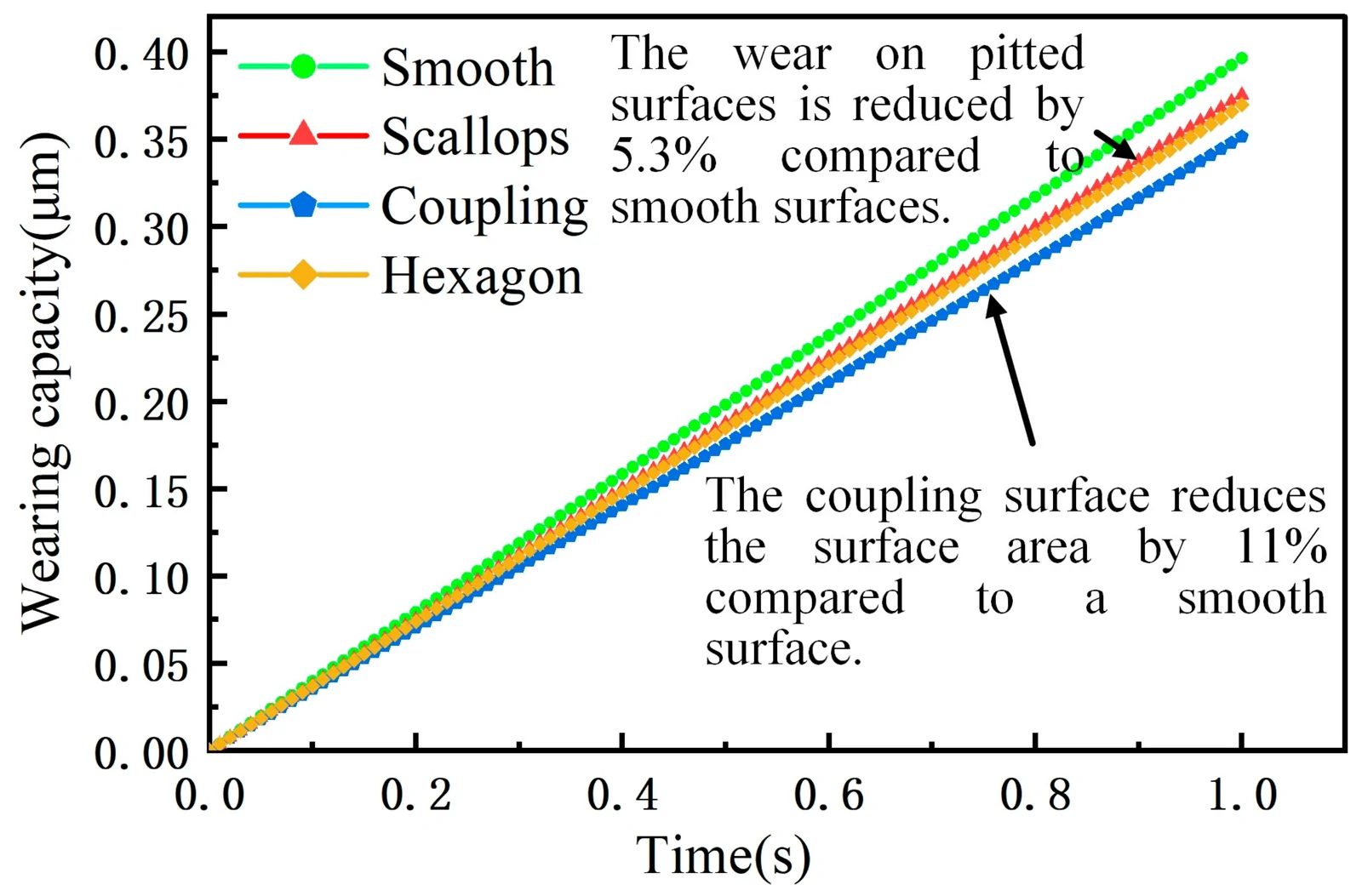
Simulated cumulative wear volume for the four bionic surface models.

Figure 8 shows the simulated wear volume for the four models. Simulated wear volumes are reported in μm^3^·mm^−1^ (cumulative wear volume per unit contact width), calculated by integrating the wear depth profile over the contact surface. These values correspond to cumulative material removal over the 8 mm simulated sliding distance. The cumulative wear volumes for S0, S1, S2, and S3 were 0.396 μm^3^, 0.375 μm^3^, 0.369 μm^3^, and 0.351 μm^3^, respectively, indicating that surface microstructure has a pronounced effect on wear performance. S0 exhibited the highest material-removal rate because contact-stress concentration caused severe plastic deformation of asperities, and trapped wear debris promoted three-body abrasion with continuous plowing of the substrate. S1 reduced wear volume by 5.3% relative to S0 through a “debris-containment effect”: the pits trapped wear debris, converting abrasive particles from active participants in wear into isolated, inactive particles and thereby reducing the proportion of abrasive wear. S2 reduced wear by distributing load uniformly across the grid edges, lowering local peak stress and consequently reducing plastic deformation depth and fatigue damage at asperities. S3 achieved the lowest wear volume among the tested conditions through the dual synergy of “stress management” and “debris management”: the hexagonal grid first reduced debris generation at the source (by suppressing plastic deformation), and the micro-pits then efficiently captured the debris that was generated, preventing secondary damage. This multi-level strategy of “reducing debris generation (hexagonal grid) plus managing generated debris (pits)” more comprehensively suppressed material removal.

It should be clarified that the Archard-based finite element model does not explicitly simulate the formation, motion, or entrapment of loose wear debris. Therefore, the proposed debris-containment mechanism is a mechanistic interpretation supported by geometric features, observed wear morphology, and experimental trends, rather than a directly simulated phenomenon. It should be noted that the 45.8% reduction in peak contact stress does not imply an equal proportional reduction in wear. According to the Archard wear law, wear volume V = K·∫σ· ds/H, meaning that wear is determined by the integral of contact stress over the entire sliding distance rather than by the peak stress alone. The 45.8% peak-stress reduction in S3 is localized to the most severe contact region, while over the full sliding distance most contact regions experience a much smaller stress reduction. Therefore, the 11.4% reduction in cumulative wear volume is physically consistent with the 45.8% reduction in peak contact stress. Quantitatively, the simulated 11.4% wear reduction for S3 relative to S0 is in reasonable agreement with the experimental 14.3% mass loss reduction for uncoated substrates, considering the geometry-only model simplifications. The rank order (S0 > S1 > S2 > S3) is identical between simulation and experiment, confirming qualitative validity.

**Figure 9:**
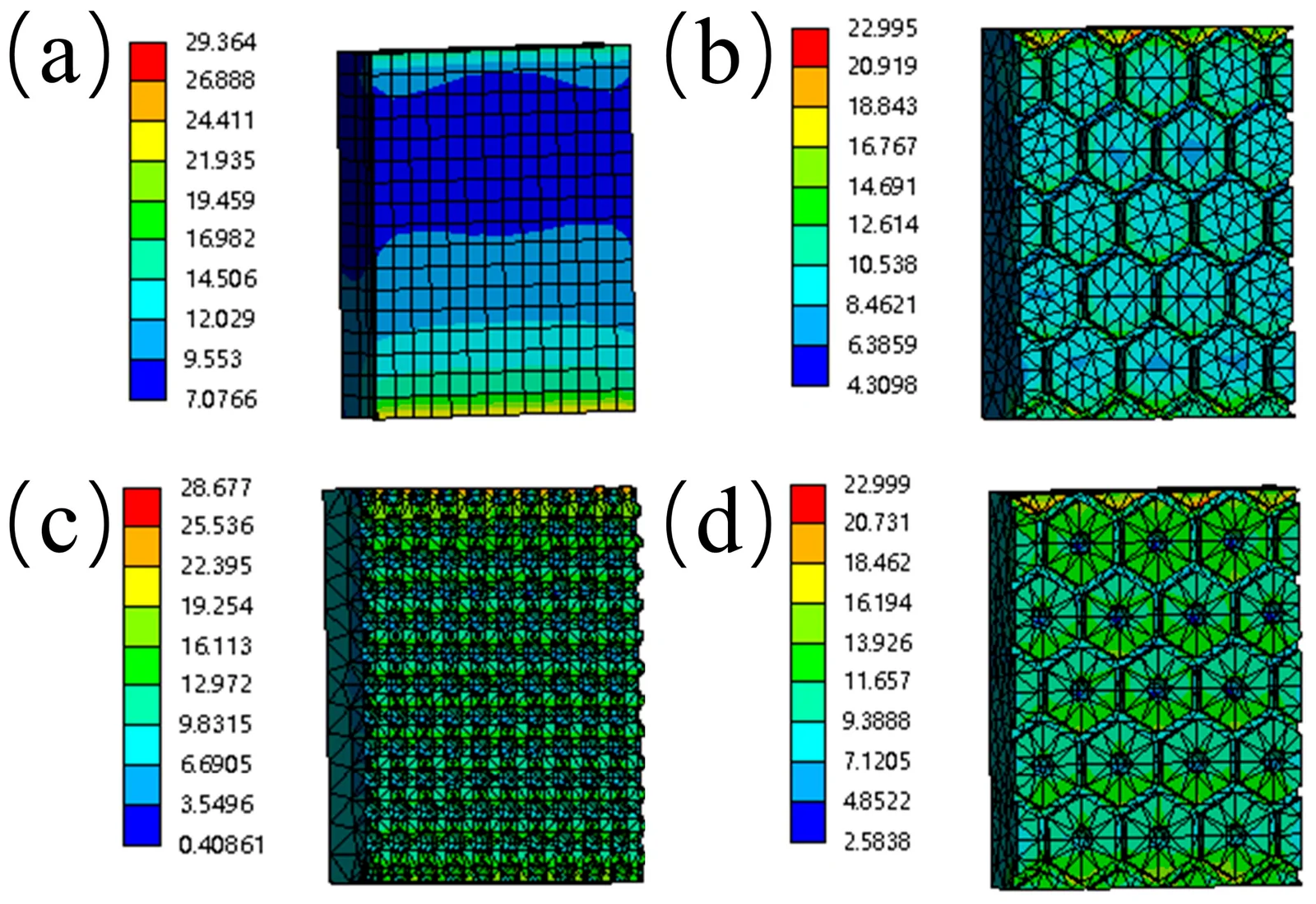
Equivalent (von Mises) stress contours: (**a**) S0 smooth; (**b**) S1 pit; (**c**) S2 hexagonal; (**d**) S3 coupled.

Figure 9 shows the equivalent (von Mises) stress distributions for the four bionic surfaces. The bionic structures produced a pronounced improvement in surface stress distribution, consistent with the frictional shear-stress results. The stable topological configuration transferred load uniformly to the grid edges, effectively avoiding local stress peaks. The equivalent stress of the coupled structure was nearly identical to that of the hexagonal structure alone, indicating that stress distribution in this simulation system was governed primarily by the macroscopic hexagonal grid. However, the microscopic debris-management function of the pits still gave the coupled structure an advantage in overall tribological performance. This result demonstrates that surface microstructure is a key factor in regulating stress concentration: rational design of surface textures (such as pits and hexagons) can effectively reduce contact stress and lower the risk of material failure.

**Figure 10:**
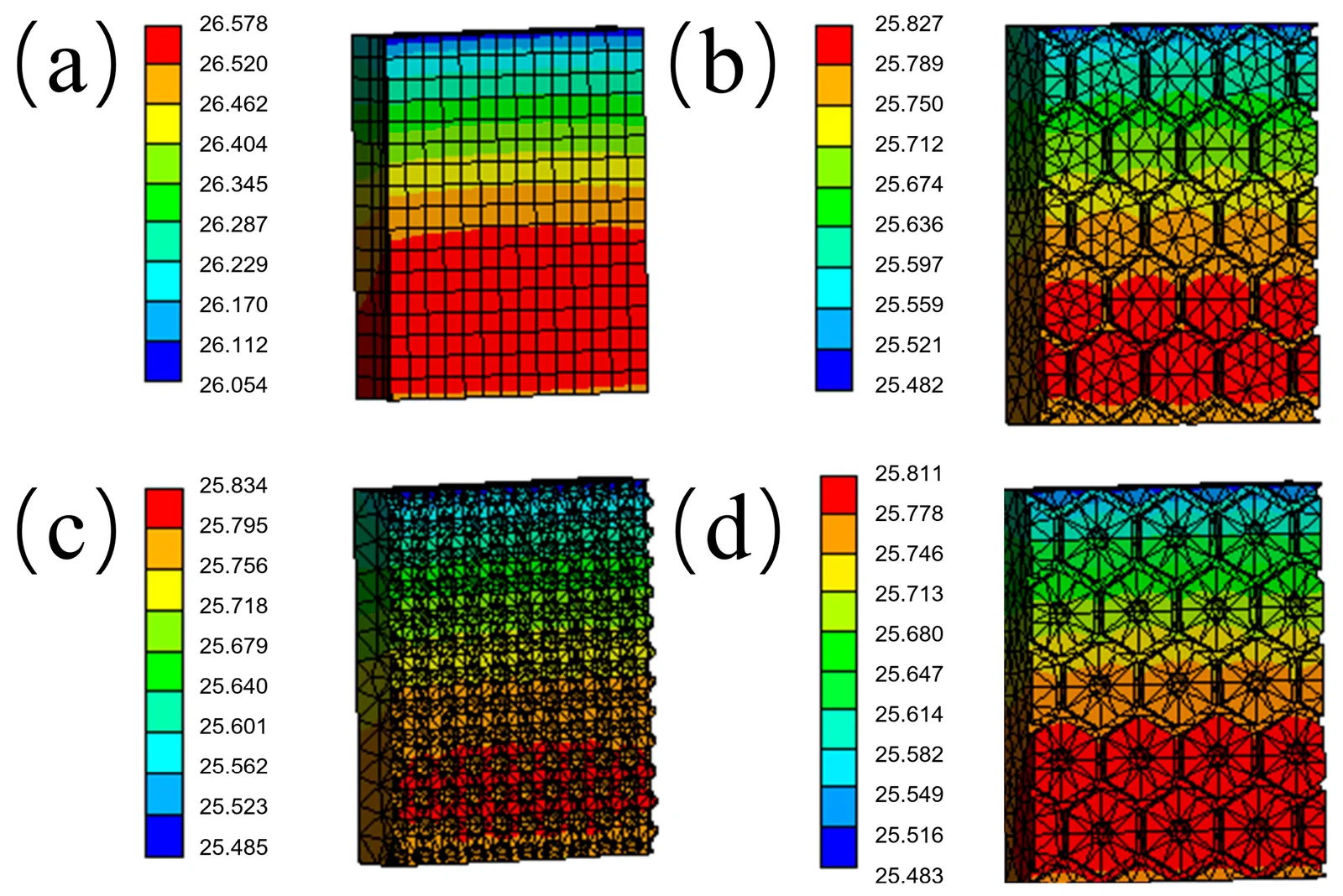
Simulated temperature distribution contours: (**a**) S0 smooth; (**b**) S1 pit; (**c**) S2 hexagonal; (**d**) S3 coupled.

Figure 10 shows the simulated temperature distributions. The maximum surface temperature of S0 was 26.578 °C, while those of S1, S2, and S3 were 25.827 °C, 25.834 °C, and 25.811 °C, respectively. All bionic surfaces suppressed frictional temperature rise. For S0, stress was highly concentrated over an extremely small contact area (Hertzian point contact), which promoted local plastic deformation, adhesive wear, or abrasive wear; meanwhile, frictional heat could not be effectively dissipated through the narrow contact region, leading to rapid local temperature rise. For S1, S2, and S3, geometric stress redistribution transferred contact pressure from sharp asperities to a broader region, lowering local contact stress and pressure per unit area and thereby reducing frictional heat generation at the source. The improved dispersion of the contact region also enhanced heat conduction efficiency, further suppressing temperature rise and indirectly reducing the wear-loss rate.

The predicted temperature difference between textures is approximately 0.7–1.6 °C above ambient and has not been experimentally validated. This modest effect suggests that frictional heating is not the dominant factor in the wear difference under the present mild sliding conditions; the primary mechanism is stress redistribution and debris management. Experimental temperature measurements will be included in future work.

**Figure 11:**
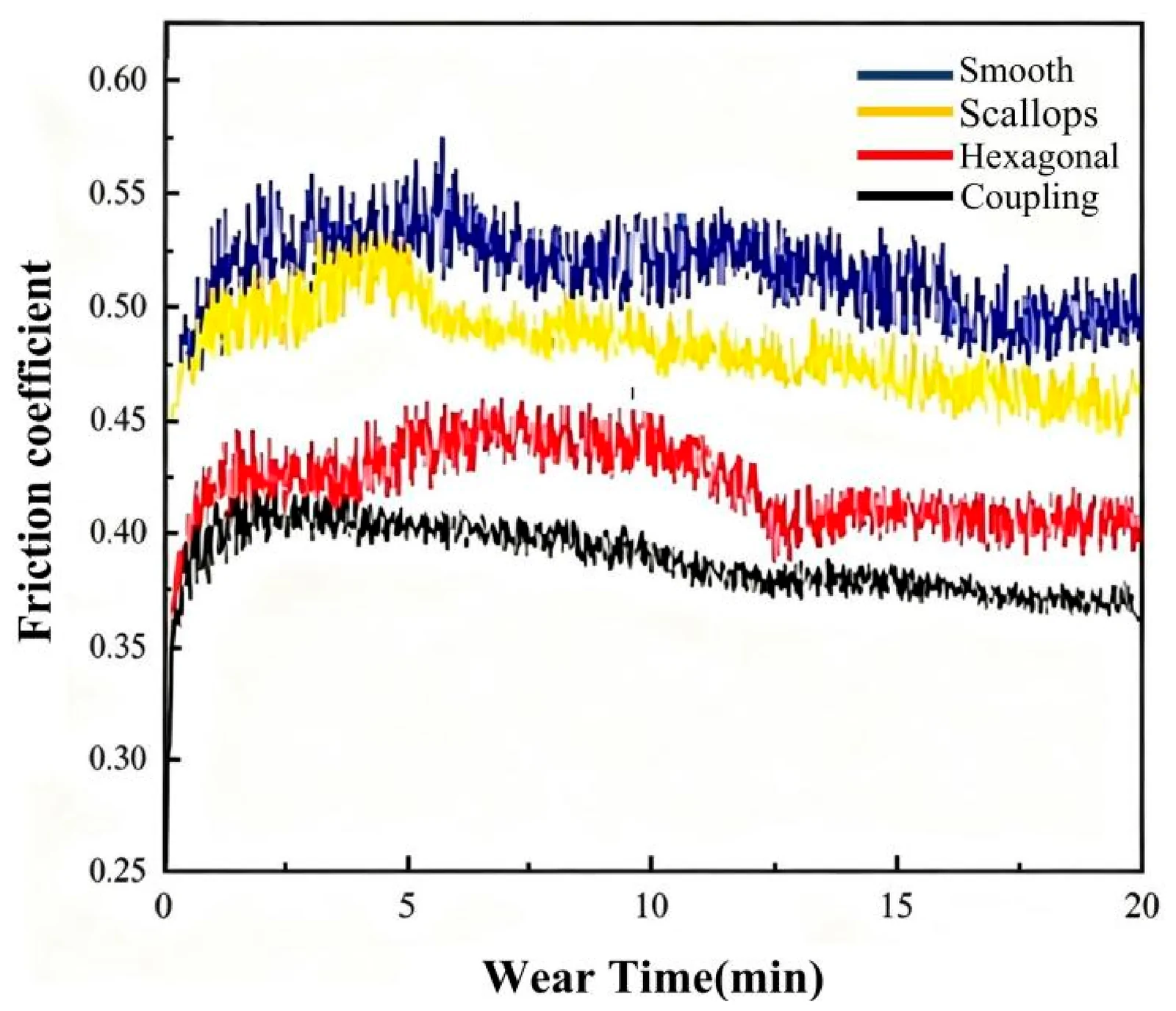
Coefficient of friction curves of different bionic substrate surfaces.

Figure 11 shows the coefficient-of-friction (COF) curves for the different bionic substrates. The average COF of S0 was 0.54 ± 0.02 with severe fluctuations, because the smooth surface provided no space for wear-debris containment, and trapped particles promoted three-body abrasion and intensified friction oscillation. S1 reduced the average COF to 0.49 ± 0.02 by capturing free wear debris in the pits. S2 further reduced the COF to 0.43 ± 0.02 by distributing contact load through the grid framework. S3 achieved the lowest average COF (0.39) among the tested conditions, combining the advantages of both structures to distribute macroscopic load and contain debris at the micro scale, with an overall stable friction curve.

**Figure 12:**
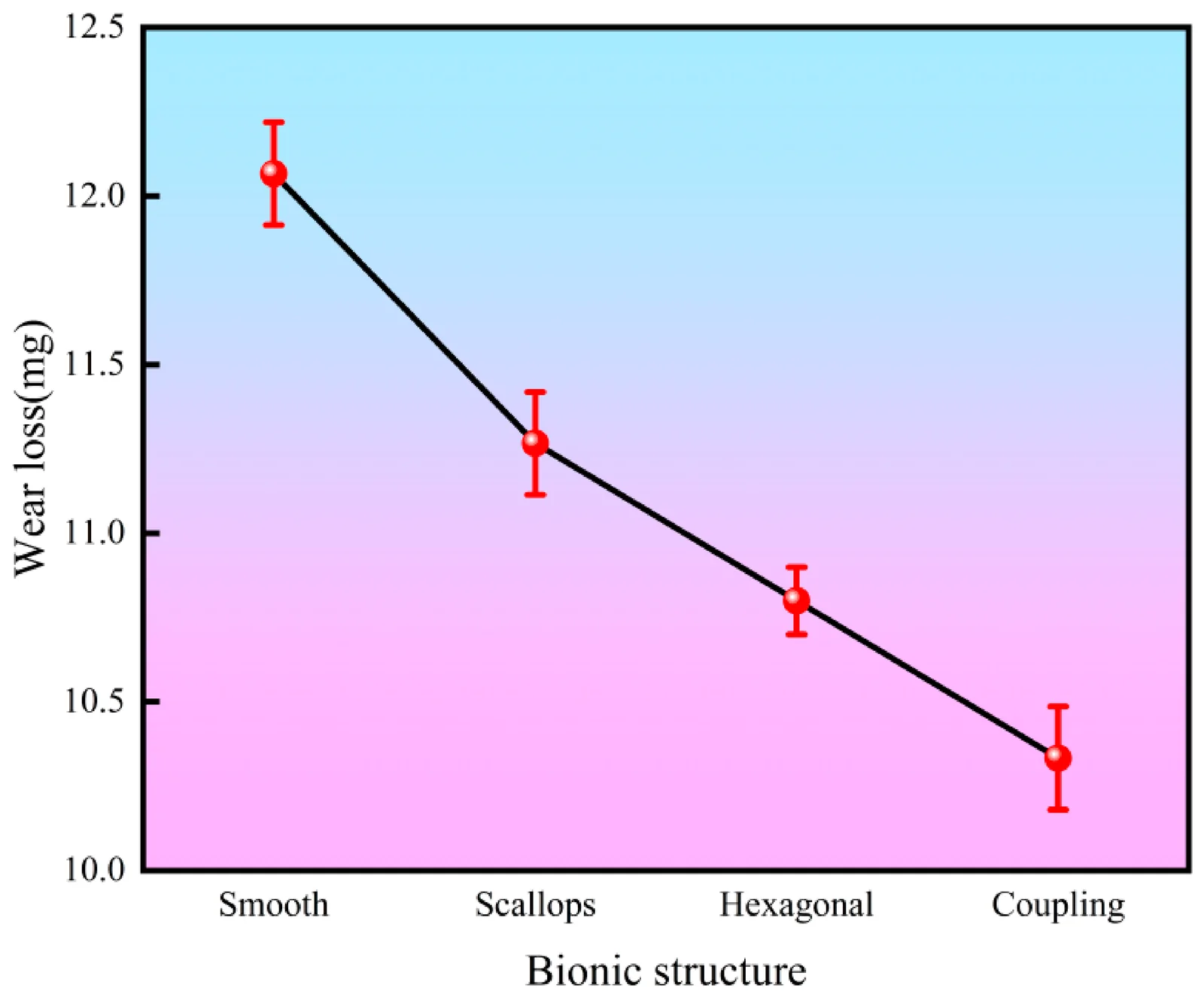
Wear mass loss of different bionic substrate surfaces. Error bars represent standard deviation (*n* = 3).

The mass loss of the different bionic substrates is shown in Figure 12. S0 exhibited the highest mass loss at 12.06 ± 0.12 mg; S1 lost 11.26 ± 0.15 mg, S2 lost 10.80 ± 0.10 mg, and S3 lost 10.33 ± 0.15 mg. The experimental trend (S0 > S1 > S2 > S3) is consistent with the simulation results, verifying at the experimental level the effectiveness of the coupled bionic structure in achieving wear strengthening through “stress distribution plus debris containment.

### 3.2. Effect of Ultrasonic Power Density on the Coatings

**Figure 13:**
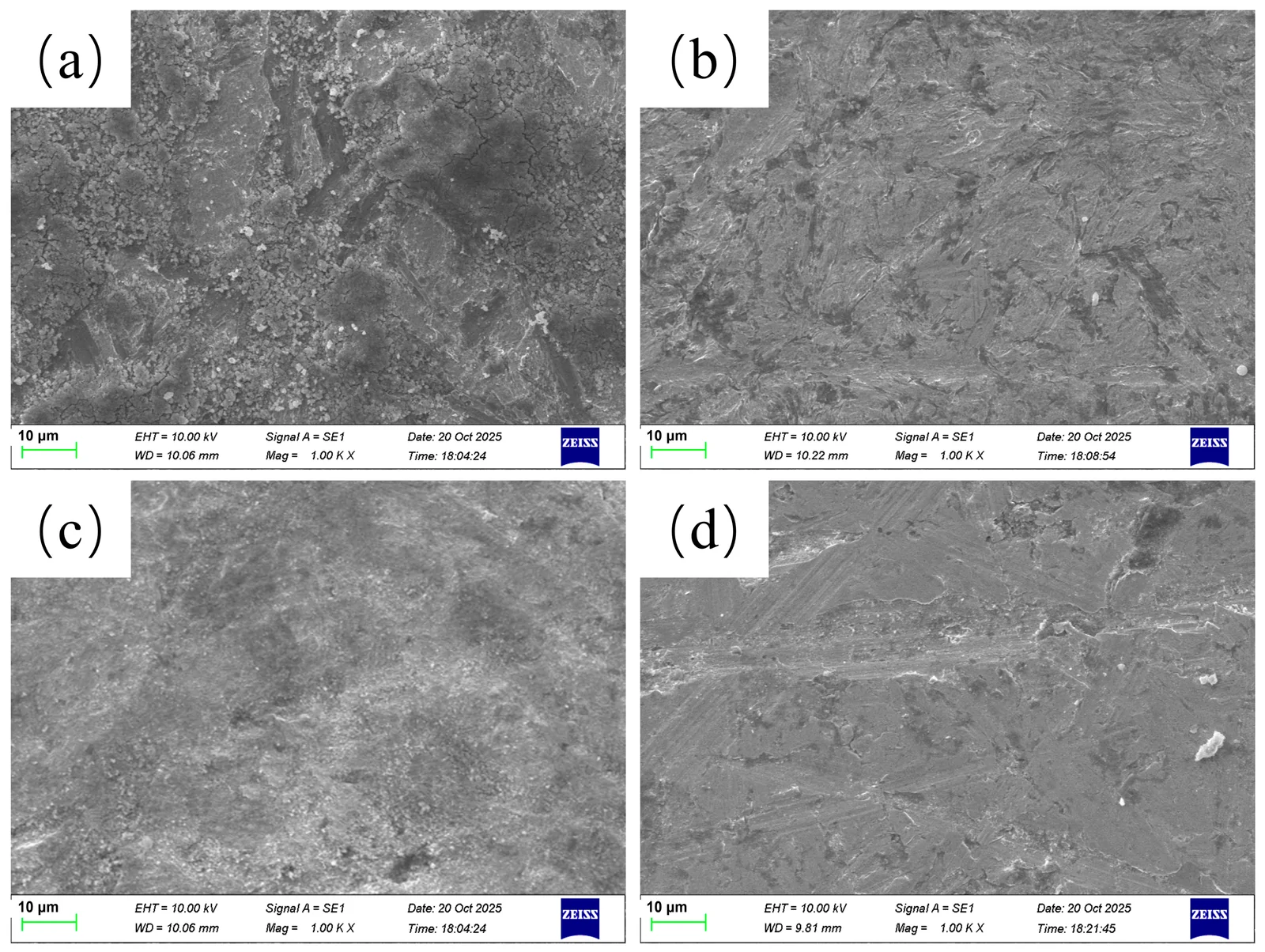
SEM surface morphologies of Ni–AlN composite coatings deposited at different UPD levels: (**a**) 0 W/cm^2^; (**b**) 15 W/cm^2^; (**c**) 25 W/cm^2^; (**d**) 35 W/cm^2^.

Figure 13 shows SEM surface morphologies of the Ni–AlN nanocomposite coatings deposited on the coupled bionic surface by ultrasonic-assisted jet electrodeposition at different UPD levels. All coatings exhibited micro–nano scale structural features, but the compactness, surface roughness, and particle-distribution uniformity differed significantly with UPD. The 0 W/cm^2^ (no ultrasound) control sample showed randomly oriented polycrystalline grains with clear intergranular gaps and prominent local grain agglomeration, indicating poor compactness. At 15 W/cm^2^, the coating exhibited a lamellar/fibrous morphology with blurred grain boundaries and improved uniformity, though minor groove defects remained. At 25 W/cm^2^, the coating showed the highest degree of grain refinement among the tested conditions, with uniform particle size, even distribution, no large grains or agglomerates, and markedly improved compactness. This improvement is attributed to the uniform dispersion of AlN nanoparticles by ultrasound at this power density, which provided abundant heterogeneous nucleation sites for metal grains [27]. The underlying mechanism is ultrasonic cavitation: the periodic collapse of cavitation bubbles generates local high temperature and pressure, strong shock waves, and microjets that enhance electrolyte mass transfer, reduce the critical nucleus size, and simultaneously break up AlN agglomerates through strong shear forces, ultimately forming a fine-grained, dense composite coating [28,29]. At 35 W/cm^2^, the coating surface exhibited scratches and mechanical damage with scattered particle impurities, indicating degraded structural integrity. Excessive ultrasonic energy induces pronounced secondary agglomeration of AlN nanoparticles, weakening grain refinement, causing grain coarsening and increased porosity, and degrading coating quality. The dispersion behavior can be further explained in conjunction with the interaction between SDS and ceramic particles. At 25 W/cm^2^, ultrasonic cavitation promoted uniform adsorption of SDS on AlN surfaces, increasing the absolute Zeta potential and making electrostatic repulsion dominant, which stabilized particle dispersion [30]. At 35 W/cm^2^, excessive energy may cause desorption or cleavage of SDS molecules, destroying the protective adsorption layer, weakening electrostatic repulsion, and increasing particle collision frequency, ultimately inducing secondary agglomeration. Quantitative image analysis of SEM micrographs (three independent 50 × 50 μm^2^ fields per sample) showed that apparent grain size decreased from 80.2 ± 4.3 nm at 0 W/cm^2^ to 34.8 ± 2.6 nm at 25 W/cm^2^, then increased to 54.6 ± 3.8 nm at 35 W/cm^2^, consistent with XRD results. Porosity fraction decreased from 2.12 ± 0.21% at 0 W/cm^2^ to 0.41 ± 0.08% at 25 W/cm^2^, then increased to 1.18 ± 0.19% at 35 W/cm^2^. Grain sizes and porosity fractions were quantified from SEM micrographs using ImageJ 1.53t (National Institutes of Health, Bethesda, MD, USA) thresholding; at 50 × 50 μm^2^ fields, the effective pixel resolution was ≈10 nm, sufficient for the reported nanoscale features.

**Figure 14:**
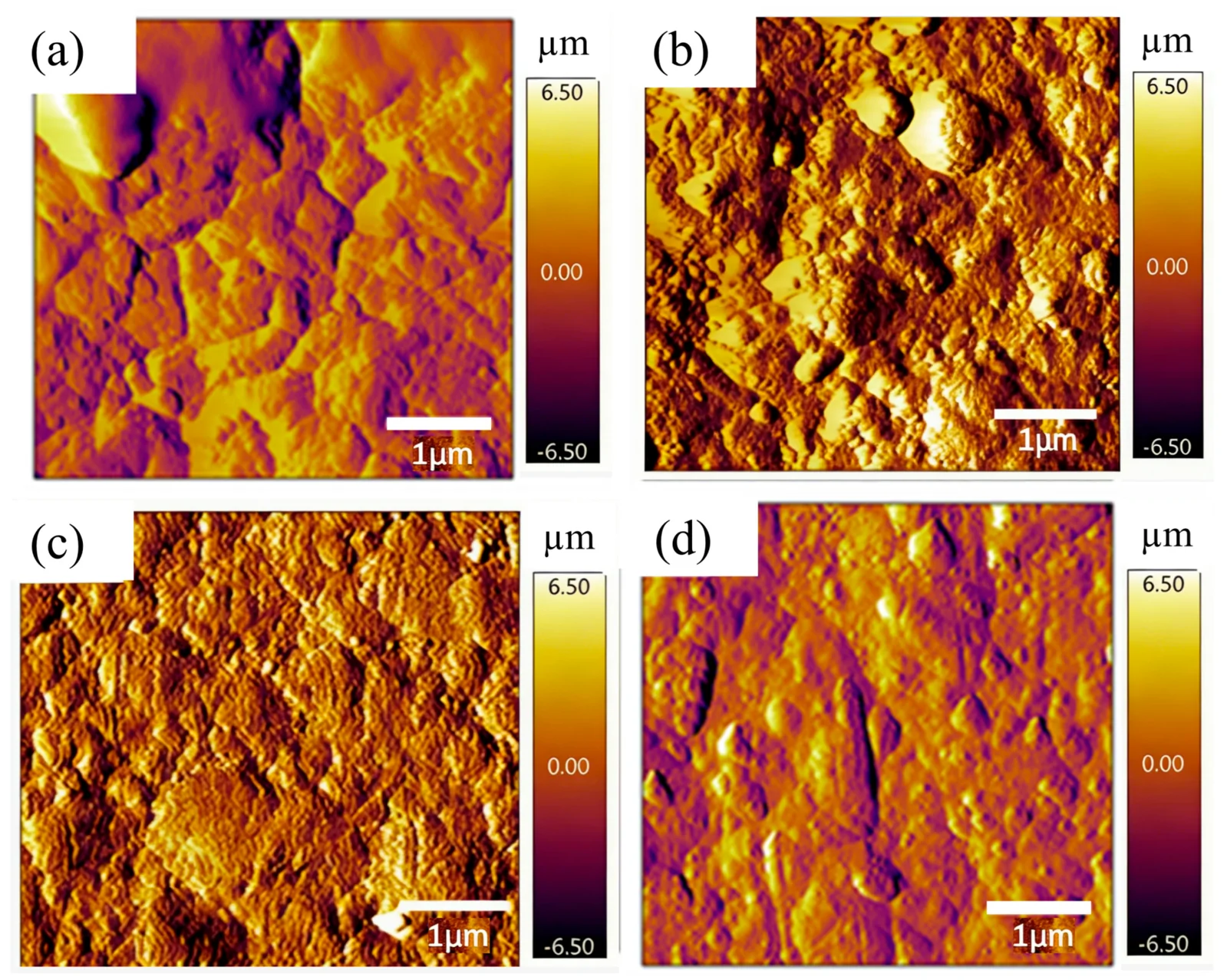
AFM surface topography of Ni–AlN composite coatings at different UPD levels: (**a**) 0 W/cm^2^; (**b**) 15 W/cm^2^; (**c**) 25 W/cm^2^; (**d**) 35 W/cm^2^.

Figure 14 shows the effect of UPD on the surface roughness of the Ni–AlN coating on the coupled bionic surface. At 0 W/cm^2^, the surface exhibited large-scale, blocky undulations with a rough, inhomogeneous morphology and significant height differences. Under no ultrasonic agitation, electrolyte mass transfer was inefficient and grain nucleation and growth were disordered, resulting in a rough, agglomerated surface. At 15 W/cm^2^, dispersed particle-like protrusions with varying sizes and heights were observed, showing a heterogeneous morphology and limited uniformity. At 25 W/cm^2^, the surface texture was fine and uniform, exhibiting a mesh-like/lamellar structure without large protrusions or depressions, indicating significant grain refinement, uniform distribution, substantially reduced roughness, and high compactness. The synergistic action of ultrasonic cavitation and acoustic streaming markedly improved electrolyte mass-transfer uniformity, promoting grain refinement and dense packing to form a uniform, fine nanoscale surface texture [31]. However, when UPD exceeded 25 W/cm^2^, surface roughness increased. At 35 W/cm^2^, uniformly distributed spherical/ellipsoidal protrusions were observed. This phenomenon arises from excessive ultrasonic energy causing violent perturbation of the electrolyte near the cathode surface, uneven local current distribution, and abnormal grain growth and protrusion defects [32]. This observation is consistent with the SEM morphological results, which showed evident molten-droplet agglomeration and irregular undulations on the coating surface under high-power ultrasound. Quantitative roughness values decreased monotonically from Ra = 0.48 ± 0.04 μm at 0 W/cm^2^ to Ra = 0.22 ± 0.02 μm at 25 W/cm^2^, then increased to Ra = 0.31 ± 0.03 μm at 35 W/cm^2^. Corresponding Rq values were 0.62 ± 0.05 μm, 0.30 ± 0.03 μm, 0.28 ± 0.02 μm, and 0.39 ± 0.03 μm, respectively. All AFM images used a consistent z-scale of 6.5 μm (note: the color-bar labels in Figure 14 are expressed in μm) for direct comparison. The lowest roughness at 25 W/cm^2^ is consistent with the densest coating morphology observed by SEM.

**Figure 15:**
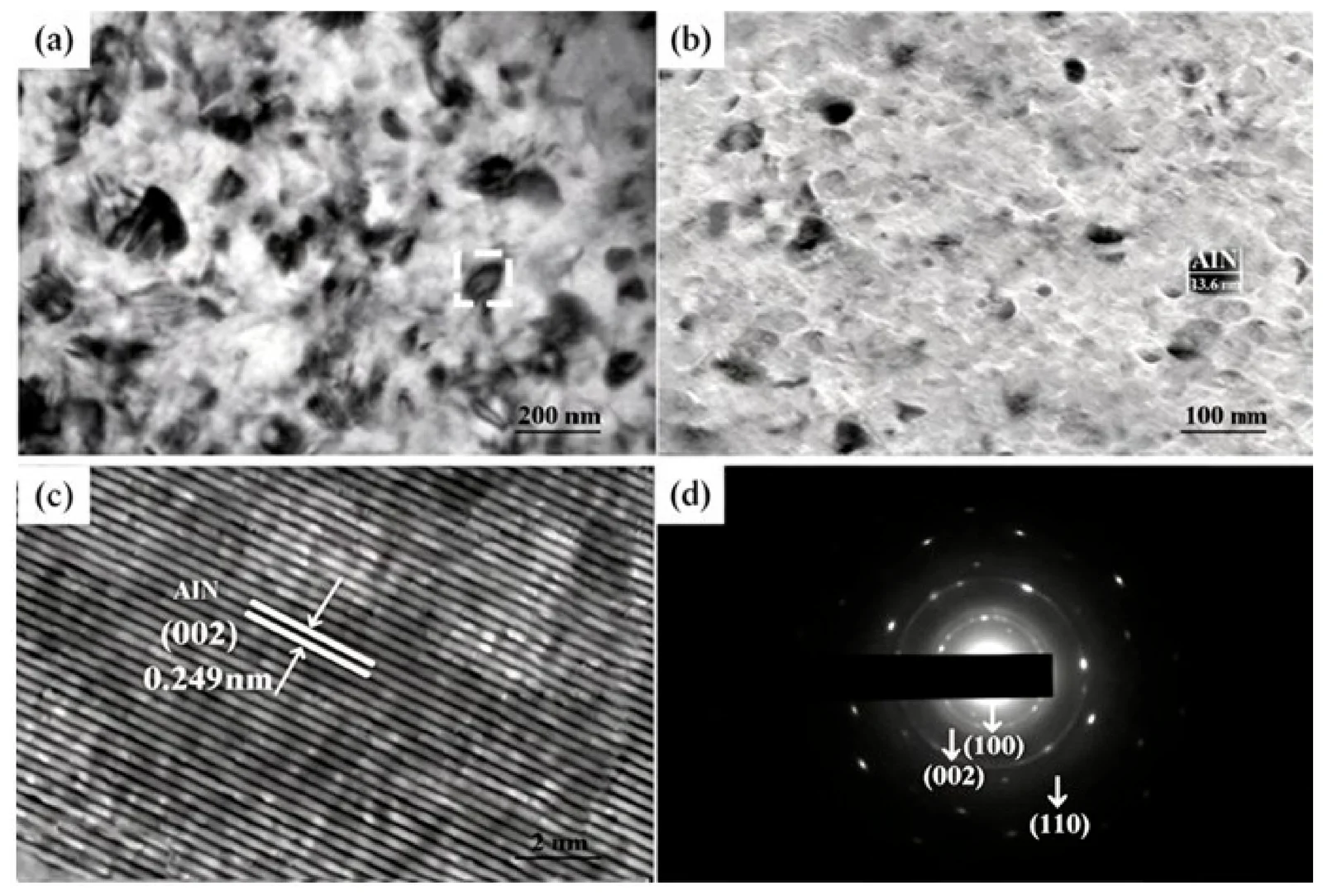
TEM images of the Ni-AlN composite coating prepared at 25 W/cm^2^: (**a**) low-magnification bright-field image; (**b**) higher-magnification image showing AlN nanoparticles; (**c**) HRTEM lattice fringes of AlN (002) plane; (**d**) selected area electron diffraction pattern.

To further investigate the microstructure of the Ni–AlN coating deposited on the coupled bionic surface at 25 W/cm^2^, TEM characterization was performed. Figure 15a shows a low-magnification TEM image revealing the overall nanoscale morphology, in which AlN grains are densely and uniformly distributed with well-developed crystalline structure. The selected region (white dashed box) clearly shows the dispersion of AlN nanoparticles within the Ni matrix, indicating highly uniform composite dispersion under this ultrasonic condition. Figure 15b shows a higher-magnification image. The mean size of the AlN nanoparticles dispersed in the Ni matrix was measured to be approximately 43.6 nm, with distinct contrast between particles and matrix confirming their crystalline nature. The composite coating exhibited an extremely dense microstructure with no observable pores or dislocation tangles. Figure 15c shows clear lattice fringes with an interplanar spacing of 0.249 nm, matching the (002) plane of hexagonal AlN and indicating that AlN nanoparticles retained their crystalline integrity during electrodeposition without significant lattice distortion. Figure 15d shows the SAED pattern; the characteristic diffraction rings correspond to the (100), (002), and (110) planes of hexagonal wurtzite AlN, confirming from a crystallographic perspective the presence of crystalline AlN nanoparticles in the Ni matrix. It should be noted that TEM characterization was performed only at 25 W/cm^2^ to confirm the coating nanostructure at the point of best performance. Direct comparative TEM across all UPD levels would be required to fully quantify the evolution of particle dispersion, and this is acknowledged as a limitation.

**Figure 16:**
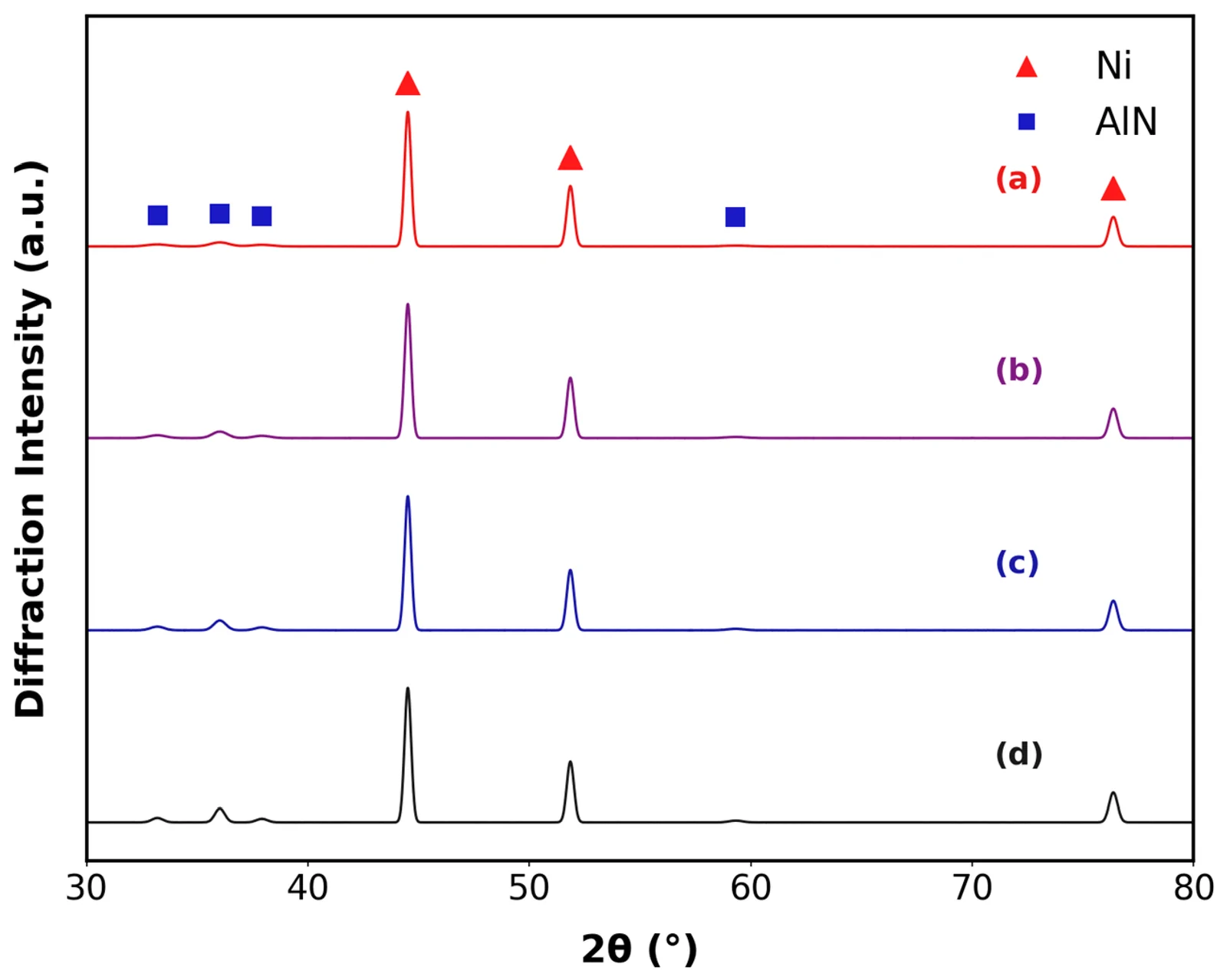
XRD patterns of Ni–AlN composite coatings prepared at different UPD levels: (a) 0 W/cm^2^; (b) 15 W/cm^2^; (c) 25 W/cm^2^; (d) 35 W/cm^2^. Red triangles mark Ni peaks; blue squares mark AlN peaks.

X-ray diffraction (XRD) was performed on the Ni–AlN composite coatings prepared on the coupled bionic surface at different UPD levels. XRD measurements were carried out on a Bruker D8 Advance diffractometer (Cu Kα radiation, λ = 0.15418 nm) in Bragg–Brentano θ–2θ geometry. Operating conditions were 40 kV and 40 mA, with a scan range of 20–90° 2θ, a step size of 0.02°, and a scanning speed of 2°/min. Raw data were processed with linear background subtraction and Kα2 stripping. No smoothing was applied to the presented patterns. Instrumental broadening was calibrated using a standard silicon powder sample (NIST SRM 640e), and the corrected FWHM was used in the Scherrer calculation with K = 0.89. The results are shown in Figure 16. Both Ni and AlN phases were identified in all coatings. The strong Ni diffraction peaks appeared at 2θ = 44.38–44.51°, 52.13–52.25°, and 76.65–76.80°, corresponding to the (111), (200), and (220) planes of face-centered cubic Ni (JCPDS No. 04-0850). AlN peaks were indexed against the wurtzite hexagonal AlN reference (ICDD PDF No. 25-1133). The measured lattice spacing of 0.249 nm for the (002) plane matches the reference value of 0.2498 nm within experimental error, confirming the wurtzite polymorph. Weak AlN diffraction peaks were observed at approximately 2θ = 33.2°, 36.0°, 37.9°, and 59.8°, corresponding to the (100), (002), (101), and (110) planes of hexagonal wurtzite AlN, respectively. These positions agree well with the reference pattern, confirming the presence of the AlN phase. The AlN diffraction peaks were weak due to the low content and dispersed distribution of AlN; their peak positions were verified by comparison with the XRD pattern of the as-received AlN powder (see Supporting Information).

Note that AlN was a pre-fabricated powder whose intrinsic particle size was fixed before deposition (mean size 43.6 nm by TEM). The Scherrer-derived AlN crystallite size represents the coherent diffraction domain size, which may be smaller than the physical particle size, as each particle may contain multiple crystallites. The apparent broadening and intensity changes in AlN diffraction peaks with increasing UPD may be related to improved dispersion of agglomerates and altered orientation distribution; however, XRD peak broadening alone cannot conclusively demonstrate dispersion improvement, as it is also influenced by microstrain and instrumental effects. This interpretation should be regarded as tentative and is supported by complementary SEM and TEM observations. To quantitatively estimate the AlN content in the coating, EDS mapping was performed on the 25 W/cm^2^ sample. The results indicate an AlN mass fraction of approximately 3–5 wt%, corresponding to a volume fraction of approximately 5–8 vol%. The diffraction-intensity ratio between Ni and AlN, estimated by the reference intensity ratio (RIR) method, is broadly consistent with the EDS results. The relatively weak AlN peaks despite their non-negligible volume fraction can be attributed to three factors: (1) the hexagonal wurtzite crystal structure of AlN has lower diffraction efficiency for some planes within the measured 2θ range compared with face-centered cubic Ni; (2) the very small AlN particle size (approximately 44 nm) causes severe peak broadening and dispersion of peak intensity; and (3) the Ni (111), (200), and (220) peaks inherently have large structure factors and high diffraction efficiency.

(9) $$D=\frac{K\lambda }{\beta \cos\theta }$$

where K is the Scherrer constant (K = 0.89), D is the average grain size (nm) perpendicular to the diffracting plane, β is the FWHM after correction for instrumental broadening (rad), λ is the incident X-ray wavelength (0.15418 nm), and θ is the diffraction angle. It should be noted that the Scherrer-derived D represents an apparent grain size that does not separate the contribution of microstrain; Williamson–Hall analysis will be used in future work to further distinguish grain size and microstrain contributions.

All four spectra exhibit the same set of AlN peaks at approximately 33.2°, 36.0°, 37.9°, and 59.8°, confirming that the wurtzite AlN phase is retained across all UPD conditions. 15 W/cm^2^, the Ni peak broadened slightly and its intensity decreased, giving a grain size of 51.4 nm. At 25 W/cm^2^, the Ni (111) peak was markedly broadened with the lowest intensity, and the Ni grain size reached a minimum of 32.5 nm. At 35 W/cm^2^, the grain size increased again to 51.3 nm. This trend is consistent with the compactness variation observed by SEM. It should be noted that the lattice parameters calculated separately from the three Ni planes ((111), (200), and (220)) showed small deviations, reflecting the anisotropic distribution of residual stress in electrodeposited coatings; Nelson–Riley extrapolation will be used in future work to obtain the stress-free lattice parameter. Figure 16 also shows that UPD significantly affected the intensity and width of Ni diffraction peaks. At 0 W/cm^2^, the Ni (111) peak was the sharpest and most intense, yielding a Scherrer-derived Ni grain size of 78.6 nm, indicating coarse grains.

Thus, UPD exerts a pronounced regulatory effect on Ni grain size in the pit-textured Ni–AlN composite coating. At 0 W/cm^2^ (no ultrasound), the coating microstructure was coarse. With increasing UPD, ultrasonic cavitation promoted uniform dispersion of AlN nanoparticles as heterogeneous nucleation sites and simultaneously suppressed Ni grain growth through Zener pinning, leading to progressive grain refinement. At 35 W/cm^2^, however, excessive ultrasonic energy disrupted particle dispersion stability and caused grain coarsening. At 25 W/cm^2^, the Ni grains were the finest (32.5 nm) among the tested conditions, providing the microstructural basis for high coating hardness and wear resistance.

**Figure 17:**
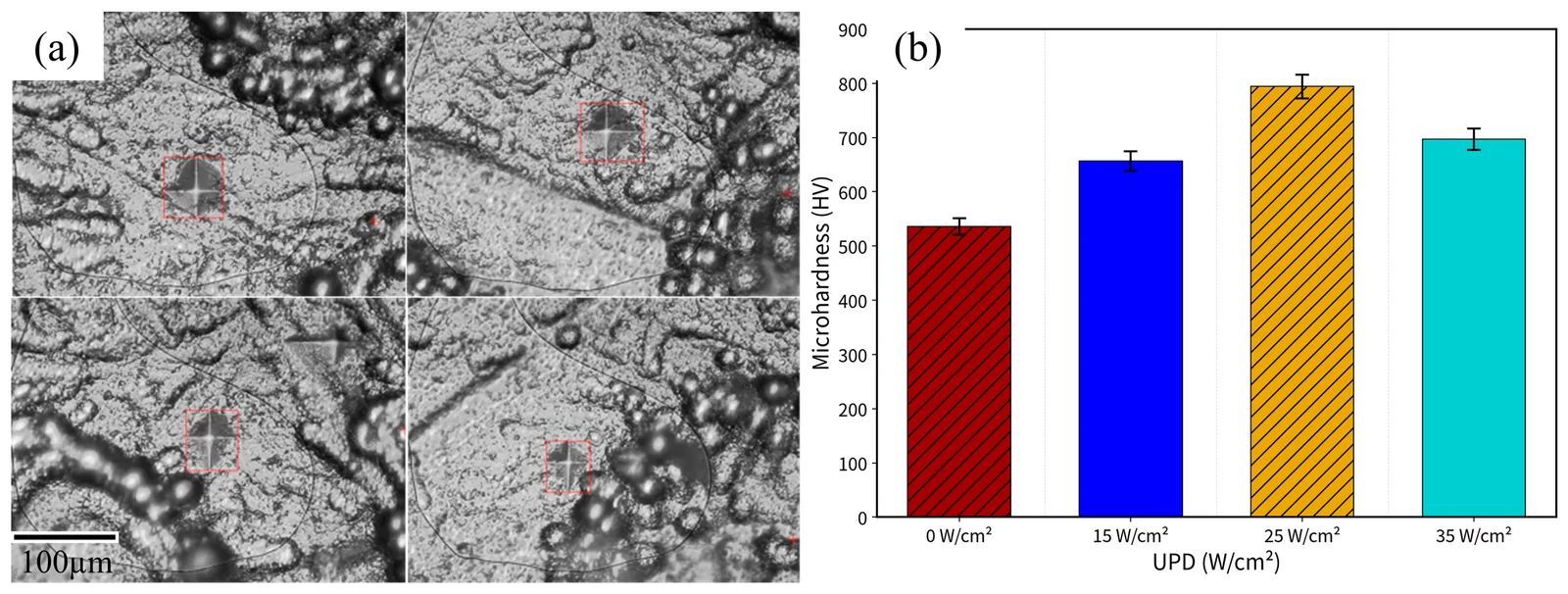
Microhardness of Ni–AlN composite coatings prepared at different UPD levels: (**a**) optical micrographs showing the Vickers indentations on the coatings deposited at 0, 15, 25, and 35 W/cm^2^; (**b**) measured microhardness. Error bars represent standard deviation (*n* = 3).

Figure 17 shows the microhardness of the composite coatings. Without ultrasound, the coating hardness was the lowest at 536 ± 15 HV. At 15 W/cm^2^, hardness increased to 657 ± 18 HV, reaching a maximum of 794 ± 22 HV at 25 W/cm^2^, and then decreased to 697 ± 20 HV at 35 W/cm^2^. The overall trend was “rise-then-fall,” consistent with reports in the literature for comparable Ni–AlN nanocomposite systems [33]. This hardness variation arises from the synergistic effect of ultrasonic cavitation and second-phase strengthening particles. On the one hand, the periodic pressure impact of cavitation can induce dislocation multiplication and grain refinement; at 25 W/cm^2^, the transient high temperature and pressure generated by cavitation-bubble collapse promoted both uniform dispersion of AlN nanoparticles and strengthened interfacial bonding with the Ni matrix. On the other hand, the hardness of the composite coating is governed by the combined contributions of grain refinement (Hall–Petch effect), Orowan bypass strengthening, and dislocation-density strengthening [34]. Uniformly dispersed nanoparticles effectively impede dislocation motion, contributing significant Orowan strengthening [35]. At 25 W/cm^2^, the highest AlN co-deposition and uniform distribution suppressed abnormal Ni grain growth; according to the Zener grain-boundary pinning theory, fine, dispersed second-phase particles effectively pin grain boundaries and inhibit grain coarsening [36]. XRD (Figure 16) shows that the Ni grain size derived from Scherrer analysis has a clear inverse relationship with microhardness (Figure 17): finer grains correspond to higher hardness. Fitting hardness (HV) against d^(−1/2)^ yielded H = 285 + 2840·d^(−1/2)^ (R^2^ = 0.96). Because coating thickness, defect density, and particle dispersion also vary with UPD, this correlation alone does not prove that grain refinement is the sole or dominant mechanism; however, it supports an important contribution of grain refinement to the observed hardness trend. The 95% confidence interval for the slope is approximately 2100–3580.

However, because AlN particle content and dispersion also varied with UPD, the Orowan contribution cannot be fully separated from the hardness–grain-size correlation alone. To further quantify the relative contributions, the Orowan equation Δσ ≈ 0.13·G·b/(r·√f) was used for a rough estimate, where G = 76 GPa (shear modulus of Ni), b = 0.249 nm (Burgers vector), r = 22 nm (half of the measured AlN particle diameter of 43.6 nm), and f ≈ 5% (volume fraction estimated from the bath concentration of 8 g/L and typical co-deposition efficiency). The calculated Orowan contribution was approximately 505 MPa at 25 W/cm^2^. At 0 W/cm^2^, severe AlN agglomeration increased the equivalent particle radius to approximately 50 nm and reduced the effective volume fraction to approximately 2%, yielding an Orowan contribution of approximately 348 MPa. The difference of approximately 157 MPa accounts for approximately 19% of the total strength improvement (ΔH = 258 HV, corresponding to Δ σ ≈ 844 MPa). This indicates that grain refinement is the dominant strengthening mechanism, while Orowan particle strengthening makes a smaller secondary contribution. It should be noted that this estimate is based on approximate assumptions regarding volume fraction and equivalent particle size; precise separation requires controlled experiments with varying AlN content at matched grain size.

**Figure 18:**
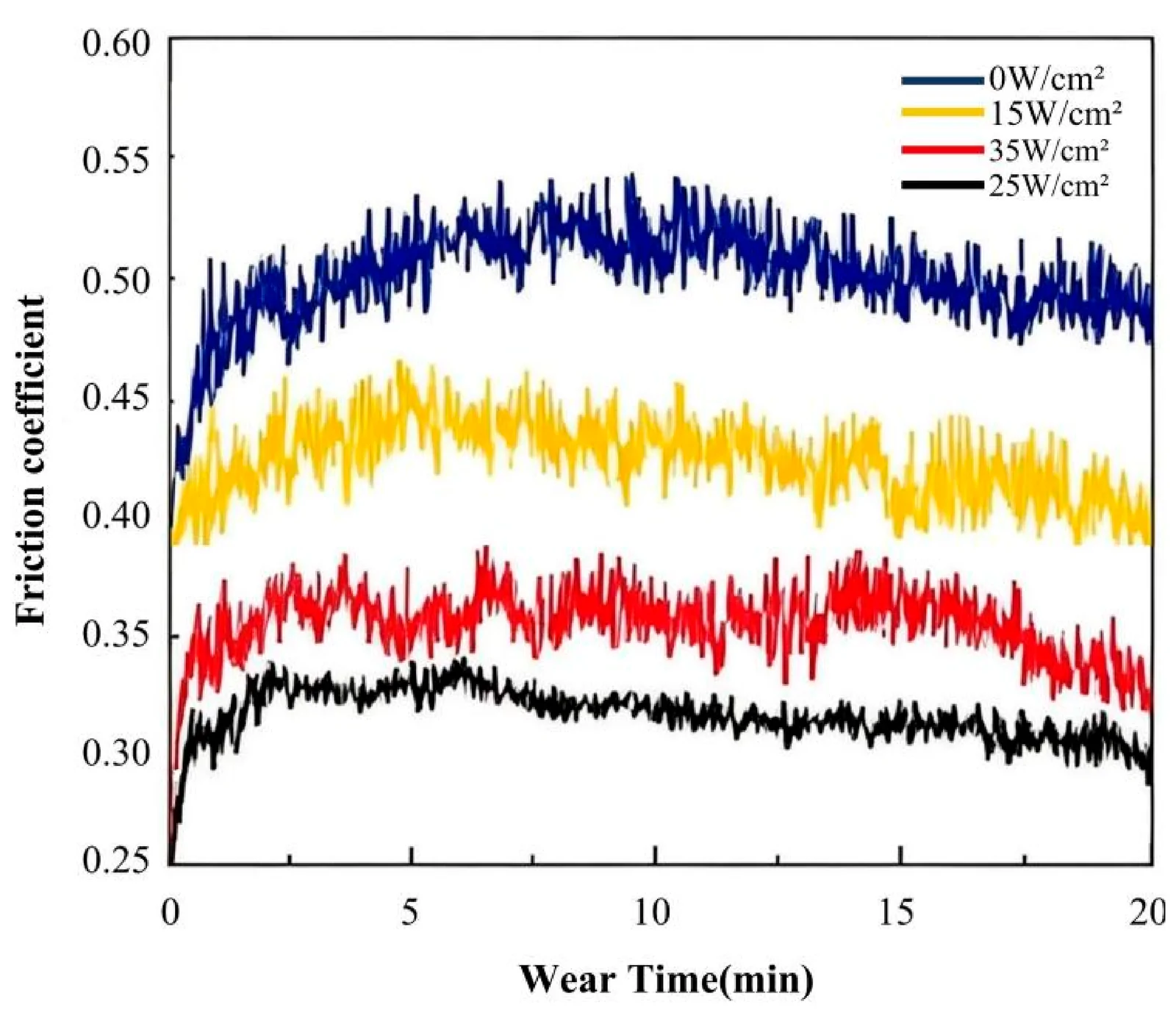
Coefficient of friction curves of Ni–AlN composite coatings prepared at different UPD levels.

Figure 18 shows the COF comparison of the composite coatings after tribological testing. The Ni–AlN coating prepared without ultrasound had the highest average COF at 0.53 ± 0.02 with severe curve fluctuations. The coating prepared at 15 W/cm^2^ had an average COF of 0.44 ± 0.02, a marked reduction indicating that the introduction of the ultrasonic field improved wear resistance. The coating prepared at 25 W/cm^2^ exhibited the lowest average COF (0.32 ± 0.01) among the tested conditions, with a stable friction curve. At 35 W/cm^2^, the COF increased to 0.36 ± 0.02 with noticeably larger amplitude fluctuations. Combined with surface-morphology analysis, the higher surface flatness and lower roughness of the 25 W/cm^2^ coating promoted stable contact during prolonged sliding, resulting in a smooth COF curve. The coatings prepared at 15 and 35 W/cm^2^ had higher surface roughness and poorer wear resistance; fracture-generated debris during sliding induced severe vibration of the tribopair, intensifying COF fluctuations. Note: The coefficient-of-friction curves show the first 20 min of sliding, by which point steady-state friction was achieved for all specimens; total test duration was 30 min.

**Figure 19:**
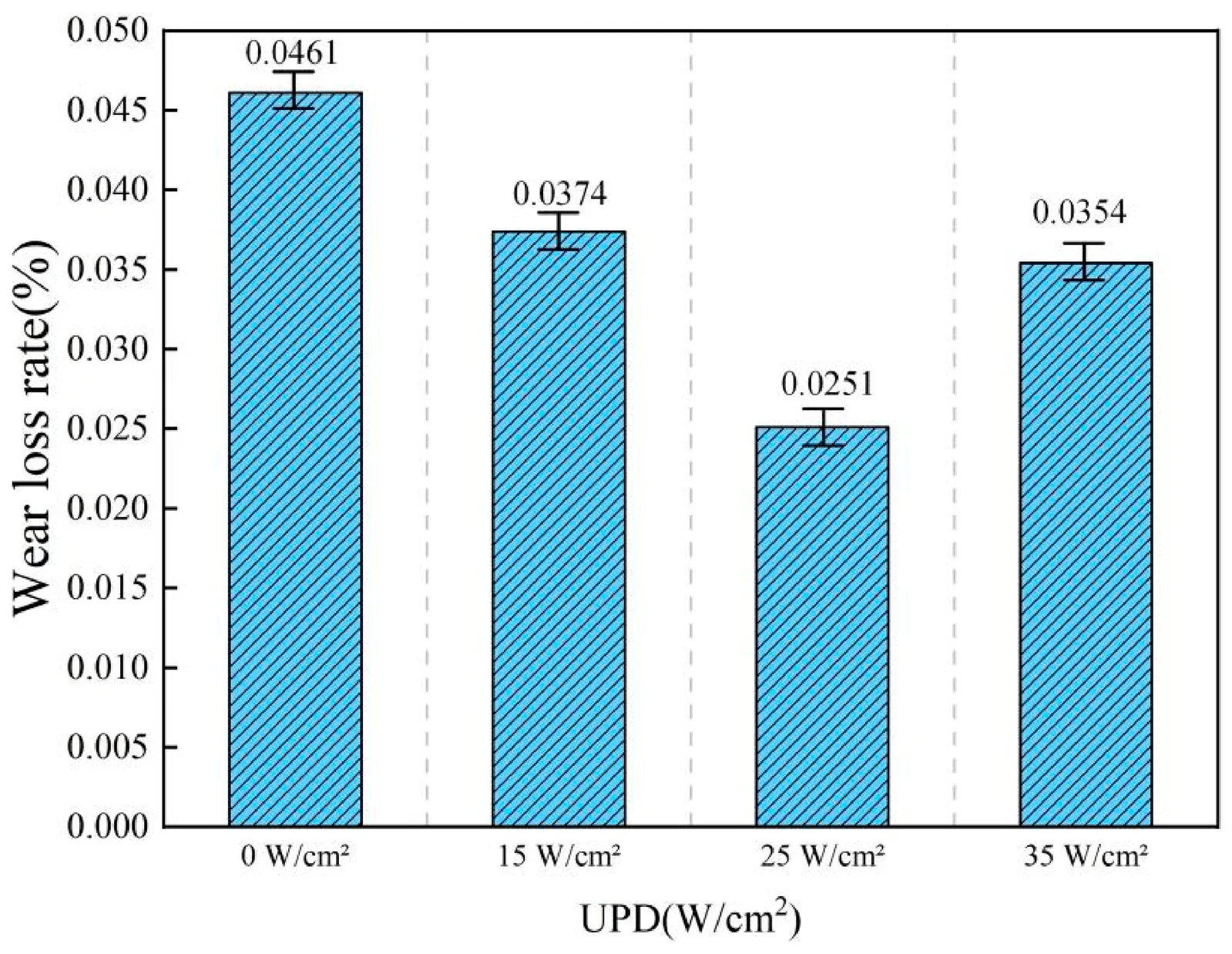
Wear-loss rate of Ni–AlN composite coatings prepared at different UPD levels. Error bars represent standard deviation (*n* = 3).

Figure 19 shows the wear-loss rate of the composite coatings, illustrating the regulatory effect of UPD on wear performance. The wear-loss rate followed a pronounced “decrease-then-increase” trend with increasing UPD, consistent with the microhardness and COF results. At 25 W/cm^2^, the wear-loss rate reached a minimum, indicating the highest wear resistance among the tested conditions. Deviation from this value in either direction caused an increase in wear-loss rate. Without ultrasound (0 W/cm^2^), the coating suffered from AlN agglomeration, coarse grains, and a porous structure, leading to particle pull-out and substrate plowing during wear and significant mass loss. Previous studies have shown that ultrasonic-assisted jet pulse electrodeposition can effectively break nanoparticle agglomerates and promote uniform dispersion in Ni–AlN nanocoatings, thereby reducing the coefficient of friction and improving wear resistance. At 35 W/cm^2^, excessive ultrasonic energy induced secondary particle agglomeration and surface mechanical damage, weakening grain refinement and second-phase strengthening and similarly degrading wear resistance. These results confirm that an appropriate UPD optimizes AlN dispersion, refines grains, and increases coating compactness, constructing a stable system of “nanoparticle reinforcement plus dense structure” that reduces material removal at the source. It should be noted that coating thickness increases with UPD (from 37.5 μm at 0 W/cm^2^ to 60.8 μm at 35 W/cm^2^), which is a confounding factor. Part of the hardness increase can be attributed to increased thickness and reduced substrate influence. However, the non-monotonic trend (peak at 25 W/cm^2^) cannot be explained by thickness alone, confirming that microstructure and particle dispersion play a dominant role.

**Figure 20:**
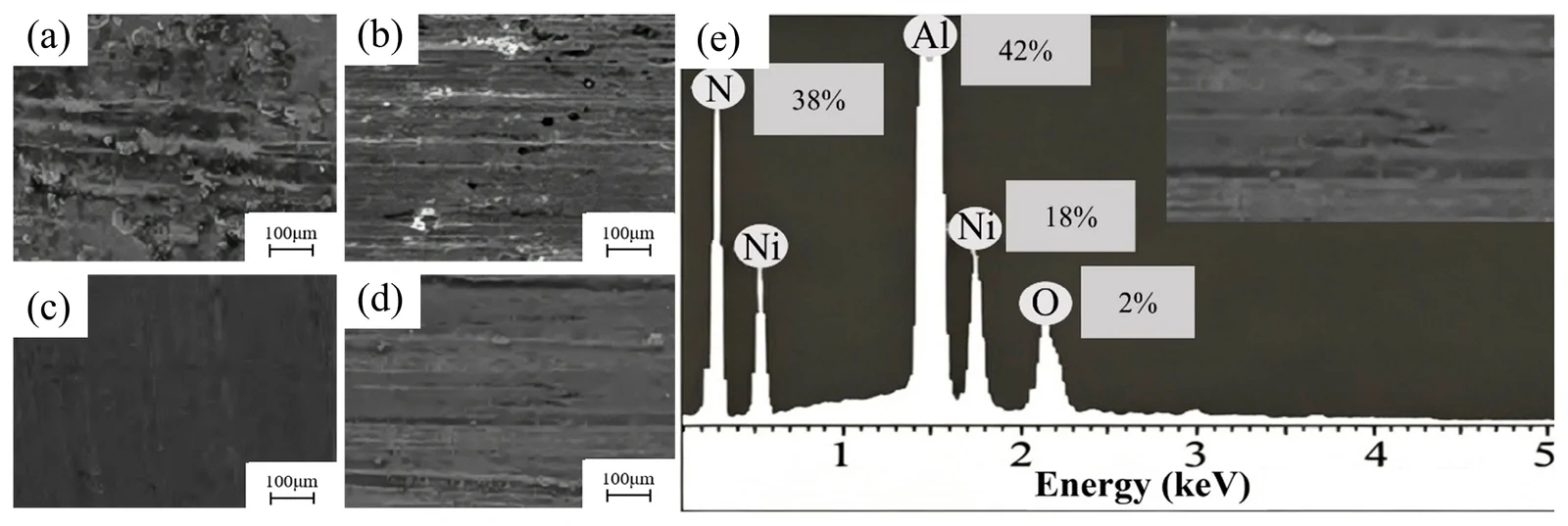
SEM morphologies of worn surfaces of Ni–AlN composite coatings at different UPD levels: (**a**) 0 W/cm^2^; (**b**) 15 W/cm^2^; (**c**) 25 W/cm^2^; (**d**) 35 W/cm^2^; (**e**) EDS spectrum of the marked particle in (**d**).

Figure 20 shows SEM images of the worn surfaces. At 0 W/cm^2^ (Figure 20a), the worn surface exhibited typical adhesive wear characteristics with large, flower-like adhesive regions and deep scratches; in some areas, the substrate was exposed, indicating severe wear. At 15 W/cm^2^ (Figure 20b), multiple fine scratches formed on the surface, indicating reduced wear. At 25 W/cm^2^ (Figure 20c), the wear track width was markedly narrowed and only very shallow scratches were visible, characteristic of mild abrasive wear. At 35 W/cm^2^ (Figure 20d), obvious grooved scratches reappeared on the surface. EDS point analysis was performed on the residual hard particle indicated by the arrow in Figure 20d (result shown in Figure 20e). The elemental composition was Al (42 at.%), N (38 at.%), Ni (18 at.%), and O (2 at.%). The Al-to-N atomic ratio of approximately 1.1:1 closely matches the stoichiometric ratio of AlN (1:1). The low Ni content indicates that this particle was not a simple protrusion of the Ni matrix but an independent phase detached or exposed from the coating. The small amount of O may be attributed to slight surface oxidation during the wear process. These EDS results are consistent with the interpretation that the hard particles observed on the wear surface at 35 W/cm^2^ are secondary agglomerates of AlN nanoparticles, providing supporting microstructural evidence that excessive UPD aggravates AlN agglomeration. It should be noted that EDS point analysis confirms the presence of Al-rich/N-rich hard particles on the worn surface; however, strictly demonstrating the reversibility of this agglomeration would require complementary dispersion-stability measurements (e.g., zeta-potential or DLS analysis before and after redispersion), which are beyond the scope of the present work.

**Figure 21:**
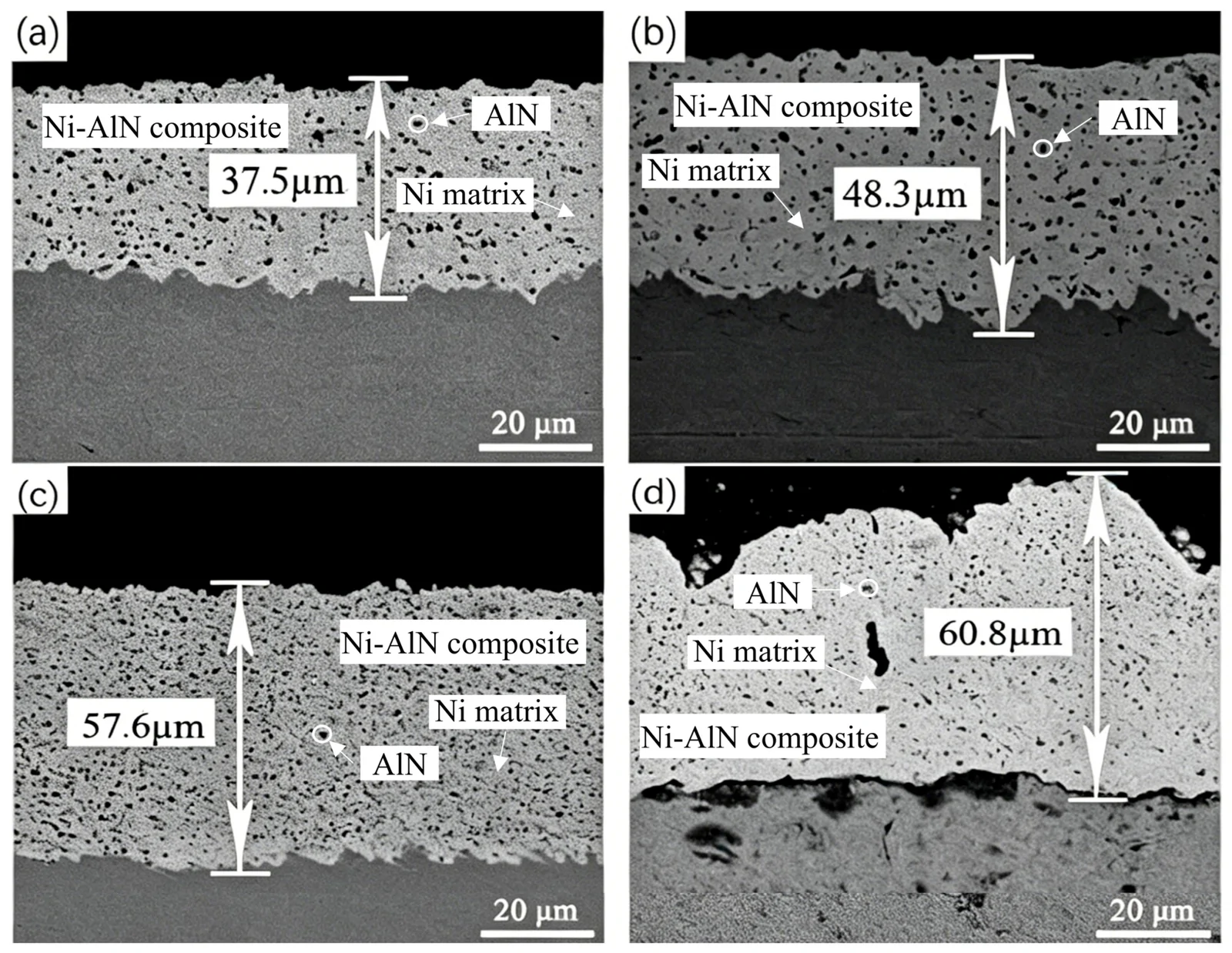
Cross-sectional SEM morphologies of Ni–AlN composite coatings at different UPD levels: (**a**) 0 W/cm^2^; (**b**) 15 W/cm^2^; (**c**) 25 W/cm^2^; (**d**) 35 W/cm^2^.

Figure 21 shows cross-sectional SEM images of the Ni–AlN coatings deposited at different UPD levels. Panels (a)–(d) correspond to UPD values of 0, 15, 25, and 35 W/cm^2^, respectively. All coatings showed no obvious cracks or pores at the interface by cross-sectional SEM, indicating good interfacial bonding quality under all UPD conditions. Direct adhesion testing will be required to quantitatively validate interfacial strength in future work. At 0 W/cm^2^, the coating thickness was approximately 37.5 μm, with numerous micropores and porous regions inside; AlN particles were distributed chaotically with local agglomeration, and the overall structure was coarse, indicating disordered grain growth and low particle co-deposition efficiency without ultrasound. At 15 W/cm^2^, the thickness increased to 48.3 μm with improved compactness, reduced porosity, and slightly improved uniformity of AlN dispersion, though minor particle accumulation was still visible locally. At 25 W/cm^2^, the coating thickness was 57.6 μm with the finest and most compact cross-sectional microstructure: grains were closely packed with no obvious pores or cracks, and AlN nanoparticles were uniformly dispersed throughout the Ni matrix without significant agglomeration. This microstructure directly reflects the synergistic effect of ultrasonic cavitation at this power density—the shock waves and microjets generated by cavitation-bubble collapse effectively break up nanoparticle agglomerates while promoting heterogeneous nucleation of Ni grains, forming a high-density, low-defect composite coating. This dense microstructure provides direct structural evidence for the high hardness and wear resistance of the coating. Ma et al. [37] reported that under the chosen ultrasonic power, Ni–W–SiC nanocomposite coatings exhibited grain refinement and significantly reduced porosity, consistent with the performance evolution observed in the present work. At 35 W/cm^2^, the coating thickness was 60.8 μm; although the coating retained some compactness, local secondary agglomeration of AlN particles and structural inhomogeneity appeared, with a small number of micropores visible in cross-section. Excessive ultrasonic energy disrupted particle dispersion stability and slightly degraded structural quality. Re-aggregation of AlN particles was observed in some regions. This distribution pattern is consistent with the surface SEM morphology, XRD grain-size results, and wear-performance analysis.

## 4. Discussion

### 4.1. Geometric Wear Reduction Mechanism of Bionic Surfaces

The coupled pit–hexagonal bionic structure achieves wear reduction through two synergistic geometric mechanisms. First, the periodic hexagonal grid distributes macroscopic contact load over multiple support edges, lowering peak contact stress by 45.8% relative to a smooth surface and reducing plastic deformation and debris generation at the source. Second, micro-pits distributed within the hexagonal cells act as debris reservoirs, trapping loose wear particles and converting them from active abrasive agents into stationary objects. This dual mechanism—reducing debris generation first, then managing generated debris—explains why the coupled structure outperforms either single texture alone. The finite element results confirm the stress redistribution effect, but they represent a geometry-only model that does not include the coating material strengthening effect. The qualitative rank order (S0 > S1 > S2 > S3) is identical between simulation and experiment, and the quantitative wear reduction values are within reasonable agreement given the model simplifications. This indicates that geometric effects are the primary contributor to the wear improvement of the textured substrates, with material effects providing additional enhancement for the coated specimens.

### 4.2. Microstructural Modulation by Ultrasonic Power Density

UPD exerts a non-monotonic effect on coating microstructure through competing mechanisms. At low-to-moderate power (0–25 W/cm^2^), ultrasonic cavitation enhances mass transfer, breaks up soft agglomerates, and promotes heterogeneous nucleation, leading to progressive grain refinement and improved particle dispersion. At 25 W/cm^2^, this effect reaches its maximum, producing the finest Ni grains (32.5 nm), the most uniform AlN distribution, and the densest coating structure. Beyond 25 W/cm^2^ (35 W/cm^2^), excessive acoustic energy disrupts the SDS adsorption layer on AlN particles, weakening electrostatic stabilization and increasing collision frequency, which leads to secondary agglomeration. This is consistent with the observed grain coarsening, increased roughness, and degraded wear performance. The proposed SDS-desorption mechanism is supported by EDS detection of AlN-rich hard particles on the worn surface, but direct confirmation via zeta-potential or DLS measurements remains to be performed in future work.

### 4.3. Comparison with Prior Studies

The performance of the optimized coating (794 HV, COF 0.32, wear rate ~0.025%) compares favorably with prior studies on similar systems. The higher hardness and lower friction in the present work are attributed to the combination of jet-enhanced mass transfer and the bionic surface texture. Compared with pure Ni coatings (~400–500 HV), the incorporation of AlN nanoparticles and ultrasonic grain refinement produces a hardness improvement of approximately 60–80%, which is consistent with the range reported in other Ni-based nanocomposite systems. The wear rate reduction relative to the 0 W/cm^2^ condition (~46%) is also comparable to literature values for optimized ultrasonic-assisted coatings.

### 4.4. Limitations and Future Work

This study has several limitations that should be acknowledged. First, only one substrate grade (AISI 1045), one AlN concentration (8 g/L), and four UPD levels (0–35 W/cm^2^) were investigated; wider parameter ranges will be explored in future work. Second, tribological tests were performed only under dry sliding at one load (5 N) and one speed (0.1 m/s); long-term durability, corrosion resistance, and performance under different loads/speeds remain to be evaluated. Third, TEM characterization was performed only at the 25 W/cm^2^ condition; comparative TEM across all UPD levels would provide more direct evidence of particle dispersion evolution. Fourth, the finite element model is a geometry-only simplification and does not capture coating material effects or debris motion. Future work will focus on: (i) wider UPD ranges and additional AlN concentrations; (ii) corrosion and long-term durability testing; (iii) direct particle dispersion characterization via zeta-potential and DLS measurements; (iv) quantitative adhesion testing; and (v) more comprehensive finite element models incorporating coating material properties and debris effects.

## 5. Conclusions

(1) The coupled bionic surface (S3) improved dry-sliding wear resistance through a synergistic mechanism of “macroscopic load-bearing by the hexagonal grid plus microscopic wear-debris management by the pits.” Finite element simulation showed that S3 reduced the peak contact stress by 45.8% and the cumulative wear volume by 11.4% relative to the smooth surface; these disproportionate reductions arise because wear is determined by the time-integrated contact stress over the sliding distance rather than by the peak stress alone. Uncoated substrate experiments confirmed that S3 achieved a lower COF (0.39 vs. 0.54) and lower mass loss (10.33 mg vs. 12.06 mg) than both the smooth surface and the single-structure bionic surfaces, with simulation and experimental trends being consistent.

(2) UPD exerted a non-monotonic (“rise-then-fall”) effect on the microstructure and overall performance of the Ni–AlN composite coating. Among the tested UPD range of 0–35 W/cm^2^, at UPD = 25 W/cm^2^, AlN nanoparticles (mean size 43.6 nm by TEM) were uniformly dispersed, Ni grains were refined to 32.5 nm, and the coating was densest among the tested conditions, corresponding to a microhardness of 794 ± 22 HV, a COF of 0.32, and a wear loss rate of approximately 0.025%. Hall–Petch fitting (R^2^ = 0.96) supports an important contribution of grain refinement, with a smaller contribution from Orowan particle strengthening; the exact quantitative partition requires further experimental separation. It should be emphasized that AlN was pre-fabricated powder whose intrinsic grain size was fixed before deposition and did not vary with UPD; the apparent broadening of AlN diffraction peaks with increasing UPD reflects improved dispersion of agglomerates rather than a change in the original grain size.

(3) Excessive ultrasonic power (35 W/cm^2^) induced pronounced secondary agglomeration of AlN particles through a hypothesized mechanism likely involving SDS desorption from AlN surfaces, loss of electrostatic repulsion, and increased particle collision frequency. Worn-surface observation and EDS analysis are consistent with the presence of AlN-rich particles (Al:N ≈ 1.1:1) on the wear surface, supporting the proposed three-body wear mechanism, shifting the wear mechanism from mild abrasive wear toward more severe three-body abrasion.

(4) This study is limited to one substrate grade, one AlN concentration, and dry sliding under a single set of test conditions. Future work will explore wider parameter ranges, corrosion performance, long-term durability, and more direct particle dispersion characterization.

## Figures and Tables

**Table 1:** Bath composition and process parameters of Ni–AlN coatings.

Item	Parameter	Function
Nickel sulfate hexahydrate	35 g/L	Main salt, providing Ni^2+^ ions
Nickel chloride	10 g/L	Anode activator, preventing nickel anode passivation
Sodium dodecyl sulfate	15 mg/L	Surfactant, improving cathode surface wettability and reducing pinhole defects
Aluminum nitride nanoparticles	8 g/L	Second-phase reinforcing particles, improving coating hardness and wear resistance
Boric acid	40 g/L	pH buffer, stabilizing the pH of the plating solution
Current density	3 A/dm^2^	—
Pulse frequency	100 kHz	—
Duty cycle	40%	—
Temperature	45 °C	—
pH value	5.5	—
Ultrasonic power density acting on cathode surface	0, 15, 25, 35 W/cm^2^	—
Electroplating time	50 min	—
Water bath heating system	500 W titanium alloy heating rod	Maintaining constant plating solution temperature (45 ± 1 °C)
Plating solution circulation system	Magnetic drive pump + φ1.5 mm nozzle	Achieving high-speed jet impact and enhancing mass transfer
Ultrasonic system	20 kHz, 0~40 W/cm^2^	Generating cavitation effect, breaking particle agglomerates and cleaning cathode surface

## Data Availability

The data that support the findings of this study are available from the corresponding author upon reasonable request. Raw XRD data files are included as Supporting Information.

## Supplementary Materials

The following supporting information can be downloaded at: https://www.mdpi.com/article/10.3390/ma19214552/s1.
